# Metformin protects against cyclophosphamide-induced ovarian fibrosis by MIF/CD74-mediated macrophage polarization

**DOI:** 10.1186/s12967-025-07294-5

**Published:** 2025-11-12

**Authors:** Ping Nie, Bo Yao, Zhejun Zhang, Jingling Li, Minghua Wang, Gendie E. Lash, Bihui Guo, Ping Li

**Affiliations:** 1 Department of Pathology, Jinan University School of Medicine, Guangzhou, 510632 China; 2 Department of Pathology, Jinan University First Affiliated Hospital, Guangzhou, 510632 China; 3 Department of Pathology, Longgang District People’s Hospital, The Second Affiliated Hospital of The Chinese University of Hong Kong, Shenzhen, 518172 China; 4 Guangzhou Institute of Pediatrics, Guangzhou Women and Children’s Medical Center, Guangzhou Medical University, Guangzhou, 510623 China; 5 Department of Obstetrics and Gynecology, Huizhou Second Maternal and Child Health Hospital, Huizhou, 516001 China

**Keywords:** MIF, CD74, Fibroblasts, Fibrosis, Premature ovarian failure

## Abstract

**Background:**

Cyclophosphamide (CTX) -induced ovarian fibrosis is involved in premature ovarian failure (POF). While metformin has demonstrated anti-fibrotic properties, the mechanism by which it regulates fibroblast activation, the primary effector cells in fibrosis, remains unclear in POF.

**Methods:**

The therapeutic effects of metformin were investigated in CTX-treated mice and further explored its interaction with macrophage-fibroblast crosstalk using an in vitro co-culture system.

**Results:**

RNA sequencing revealed that metformin suppressed the MIF/CD74 signaling pathway, which was significantly activated by CTX in ovarian tissues. In vitro, CTX increased the CD86+/CD206 + macrophage ratio via NF-κB pathway activation, indicating altered macrophage polarization. Metformin or the MIF inhibitor ISO-1 reversed this polarization imbalance, thereby attenuating fibroblast activation and extracellular matrix (ECM) production in co-culture models. Additionally, CD74 knockdown in fibroblasts downregulated ECM-related genes and inhibited MAPK/JNK signaling, whereas CD74 overexpression exacerbated the fibrotic responses.

**Conclusion:**

These findings highlight a novel mechanism by which metformin alleviates CTX-induced ovarian fibrosis by targeting the MIF/CD74 axis to reprogram macrophage-fibroblast communication, suggesting metformin as a protective adjuvant to improve ovarian health during chemotherapy.

**Supplementary Information:**

The online version contains supplementary material available at 10.1186/s12967-025-07294-5.

## Background

Cyclophosphamide (CTX) is a widely used chemotherapeutic agent for the treatment of female cancers, but it is often associated with a decline in ovarian reserve function, potentially leading to premature ovarian failure (POF) [1]. POF affects approximately 3–7% of women of reproductive age and is a significant infertility concern, particularly for young cancer patients following chemotherapy [2, 3]. Ovarian fibrosis, characterized by fibrotic changes in the ovarian stroma, has been well-documented in both human and animal models of CTX-induced ovarian dysfunction [3]. In ovarian fibrosis, fibroblasts, particularly stromal fibroblasts, play a central role in the excessive deposition of extracellular matrix (ECM) components such as collagen, which leads to ovarian dysfunction [4, 5]. However, the key regulators of fibroblast activity in CTX-induced ovarian fibrosis remain to be fully elucidated.

Macrophages are abundant and considered to regulate granulosa cell (GC) function and follicular growth and affect ovarian function [6, 7]. Moreover, macrophages are implicated in fibrotic processes in both aging ovaries and CTX-induced ovarian dysfunction [5, 8, 9]. Macrophage migration inhibitory factor (MIF), a soluble proinflammatory cytokine primarily released by macrophages, plays a pivotal role in the progression of fibrosis across various tissues through its interaction with CD74 [10, 11]. This interaction induces ECM production, which is synchronized with the upregulation of transforming growth factor-β1 (TGF-β1) in fibroblasts, contributing to tissue fibrosis [12, 13]. MIF is also expressed in the ovary and has been shown to aid in ovarian function recovery in autoimmune ovarian diseases [14]. Recent studies indicate that the NF-nuclear factor kappa B (NF-κB) pathway, which is involved in CTX-induced POF, can be activated by elevated MIF levels in the ovary [15, 16]. However, it remains unclear whether the MIF-CD74 signaling pathway regulates fibroblast activation and ECM deposition during CTX-induced ovarian fibrosis.

Metformin (N, N-dimethylbiguanide), the most widely prescribed anti-diabetic medication, has gained attention for its potential as an anti-cancer agent, demonstrating efficacy in combination with various chemotherapeutic drugs in different cancer types [17]. Notably, metformin has shown anti-inflammatory, antioxidant, and anti-apoptotic effects on GCs in vitro, as well as in CTX- or cisplatin-induced POF models, and is increasingly being explored for its potential to prevent or treat POF [18]. In animal models, such as aged or high-fat diet mice, metformin has been shown to attenuate ovarian fibrosis by modulating macrophage activity and influencing profibrotic and inflammatory signaling pathways [9, 18, 19]. While metformin has demonstrated anti-fibrotic properties, the mechanism by which it regulates fibroblast activation, the primary effector cells in fibrosis, remains unclear in POF.

CTX-induced ovarian fibrosis is a major contributor to premature ovarian insufficiency, yet the underlying mechanisms involving macrophage polarization and stromal cell interactions remain poorly defined. The current study aimed to investigate whether metformin attenuates CTX-induced ovarian fibrosis by modulating macrophage polarization and macrophage-fibroblast crosstalk, with a specific focus on the MIF/CD74 pathway. RNA sequencing (RNA-seq) of ovaries from CTX-treated mice was used to identify dysregulated pathways. Additionally, the effects of metformin on CTX-treated ovaries, 4-hydroperoxycyclophosphamide (4HC)-treated THP-1 cells (a human macrophage line), and HSF cells (a human immortalized skin fibroblast cell line) co-cultured with THP-1 cells were investigated. This integrated approach allowed us to dissect the mechanism by which metformin modulates macrophage-fibroblast crosstalk during ovarian fibrosis.

## Methods

### In vivo xenograft models

Female C57BL/6J mice (5‒8 weeks; 18.1 ± 0.1 g) were obtained from Zhejiang Vital River Laboratory Animal Technology Co., Ltd. The mice were maintained at a constant temperature of 25 °C, 35%–75% humidity and a 12:12 light/dark cycle. POF was induced by intraperitoneally injecting CTX (50 mg/kg) for 14 days. After successful modeling, the mice were randomly divided into three groups: the control group (saline-treated group), the CTX group (50 mg/kg CTX-treated group) and the metformin group (50 mg/kg CTX + 200 mg/kg metformin -treated group). All the groups were treated via i.p. injection every other day for 14 days (*n* = 20 mice/group). Uteri were obtained from mice as previously reported [20]. Body weight was measured every day, and ovarian weights were measured before each administration. All procedures complied with ARRIVE 2.0 guidelines. The completed checklist is provided as Supplementary Table S1.

The estrus cycle of the mice was monitored continuously for 28 days via vaginal smears. The vaginal samples were stained with Crystal Violet Staining Solution (Beyotime, China). The estrous cycle stage was determined according to the cell types observed under a light microscope.

### RNA sequencing and analysis

Total RNA isolated from the ovarian tissues of mice in the control, CTX and metformin groups (*n* = 3 mice per group; age, postnatal Day 80) was used to construct libraries (BGI, Shenzhen, China). The sequencing libraries were used for cluster generation and sequencing on the MGIseq2000 platform (BGI). All samples passed quality thresholds of > 75% sequences aligned and > 15 million aligned reads per sample. Differentially expressed genes (DEGs) were filtered as those with a fold change ≥ 2 and a *P* < 0.05 using the R package DESeq2 version 1.34.0. The biological functions of the DEGs were further analyzed by determining the significantly enriched Gene Ontology terms, and a pathway analysis was conducted using the Kyoto Encyclopedia of Genes and Genomes (KEGG). The RNA-sequencing (RNA-seq) data have been deposited to National Center for Biotechnology Information (NCBI) under the BioProject number PRJNA1232614.

### Hormone assays

Serum samples were obtained via the eyeball vein, and collected after centrifugation at 3000 rpm; 4 °C for 15 min. Enzyme-linked immunosorbent assay (ELISA) kits were used to measure the level of follicle-stimulating hormone (FSH) and estradiol (E2) in the serum samples (CEA830Ra, USCN Life Sciences Inc., Wuhan, China; CEA461Ge, USCN Life Sciences Inc.). The sensitivities of the FSH and E2 assays were 0.92 ng/ml 45.9 pg/ml, respectively.

### Histology assessment

The ovaries were fixed, embedded in paraffin, sectioned (5 μm thick) and stained with hematoxylin and eosin (H&E) or immunohistochemistry. The follicles in the ovaries were characterized as primordial, primary, secondary or antral follicles according to the morphologic criteria of immunohistochemistry and/or immunofluorescence stained sections as previously described [21]. The antibodies used are presented in Table S2. For Immunofluorescence (IF) experiments, sections were initially treated as for immunohistochemical analysis. After blocking with Tris-buffered saline containing 5% bovine serum albumin (BSA) for 2 h, the slides were incubated with primary antibodies at 4 °C overnight. After washing, the slides were incubated with Alexa Fluor 555-labeled goat anti-rabbit IgG or Alexa Fluor 488-labeled goat anti-mouse IgG for 2 h, and DAPI was used to label the nuclei. Antibodies are presented in Additional file: Table S2. Image acquisition was performed using a laser scanning confocal microscope (Nikon, Japan). Expression of AMH and FSHR was detected by immunohistochemical analysis. The intensity of GCs was multiplied by the percentage of positive cells to achieve a score between 0 and 12, using semiquantitative analysis with immunohistochemical reactive scores (IRSs) [21]. The percentage of positive cells was determined via observation of five randomly selected high-power fields (× 400) on each histological section. For other antibodies stained in stroma or specially stained in macrophages, immunofluorescence image analysis was performed with Image J software.

Fibrosis was indicated by areas positively stained with Masson’s trichrome (Masson staining) and Sirius Red [13]. Fibrin deposition are evaluated using Image J and the results are expressed as stained area ratio of fibrin deposition in the ovary. Photographs were taken using an epifluorescence microscope (Olympus IX51, Leica DM 4000B). All slides were assessed by two observers and employed standardized criteria for image selection and quantification. Every fifth and sixth histological section were selected for comparison and evaluation (*n* = 6 ovaries per group).

### Cell culture and reagents

The THP-1 and human granulosa cells (KGN cells) were obtained from ProCell Corporation (Wuhan, China) and cultured in RPMI 1640 medium and DMEM/F12 (Gibco, Massachusetts, USA), respectively. Human skin fibroblasts (HSF) were purchased from iCell (Shanghai, China) and cultured with DMEM medium (Gibco, Massachusetts, USA). The cell lines were supplemented with 10% fetal bovine serum (Gibco, USA) (5% CO_2_, 20% O_2_, 37 °C). The MIF antagonist, ISO-1 (HY-16692, MedChemExpress, New Jersey, USA) and the 4HC chemotherapy reagent (19527, Cayman, USA) were also used to treat cells.

For cell transfection, small interfering RNA (siRNA) was synthesized by Guangzhou Ribobo Co., Ltd (Guangzhou, China). Adenoviral vector carrying CD74 gene and encoding mNeonGreen protein was conducted by OBiO Technology Corp., Ltd., Shanghai, China. HSF cells were transfected with Ad- or empty vector with 1 × 10^5^ pfu/ml for 24 h, and were observed with a fluorescence microscope to evaluate the transfection efficiency.

For coculture, 0.4 μm transwell inserts (Corning, NY, USA) were used. A total of 5 × 10^4^ KGN cells/ml was seeded into the upper chambers of a 96-well plate, and 2 × 10^4^ HSF cells/ml was seeded into the lower chambers of a 96-well plate.

### Cell viability assay

Cell viability was determined using a CCK-8 kit (Beyotime Biotech, Nanjing, China). Briefly, cells (1 × 10^5^ cells/well) were incubated with metformin in 96-well plates for 24 h and 48 h. Quantification was performed by measuring the absorbance at 450 nm on a microplate reader (SpectroAma™ 250, Winooski, USA). The data are presented as percentages of the control.

### RT‒qPCR and Western blot analysis

RT–qPCR and western blot were performed as previously described [21]. The specific primers are presented in Additional file: Table S3. Briefly, total RNA was isolated using an RNA Extraction Kit (Takara, Japan). Synthesis of cDNA by reverse transcription was performed using a PrimeScriptTM RT Reagent Kit with gDNA Eraser (Takara, Japan). The gene expression was assessed as cycle threshold (CT) values and quantified using the Bio-Rad CFX-Connect™ real-time PCR system (Bio-Rad, USA). The relative expression of genes was normalized to β-actin. The RT-PCR results are representative of three independent experiments.

Western blotting was executed with standardized steps, using specific recognition of antibodies (Additional file: Table. S2). Proteins were extracted from cells using RIPA lysis buffer, and protein concentrations were quantified using a protein assay involving bovine serum albumin (BSA). Protein samples from different groups were separated by SDS-PAGE and transferred to polyvinylidene fluoride (PVDF) membranes (Millipore, USA). The membrane was blocked by incubating with 5% non-fat milk at room temperature for 2 h, followed by overnight incubation at 4 °C with the primary antibody specific to the target. The membranes were incubated with the appropriate peroxidase-conjugated secondary antibody for 2 h. Protein bands were visualized using an ECL kit (Thermo Fisher, USA). Greyscale band intensities were analyzed using a BioSpectrum 600 imaging system (Bio-Rad, USA) and normalized to β-actin. Western blotting results are representative of three independent experiments.

### Molecular docking

The 2D structure of the small molecule ligand was obtained by PubChem database (http://pubchem.ncbi.nlm.nih.gov/), and the 2D structure was input into Chem Office 20.0 software to make its 3D structure and saved as a mol2 file. Then the RCSB PDB database (http://www.rcsb.org/) was applied to screen the crystal structure of the protein target with high resolution as the molecular pair acceptor, and the PyMOL 2.6.0 software was used to dehydrate and dephosphorylate the proteins, and saved as a PDB file. Molecular Operating Environment 2019 software was used to minimize the energy of the compounds, preprocess the target proteins and find the active pockets. Finally, MOE 2019 was run for molecular docking with the number of operations set to 50. The binding activity of both was evaluated according to the magnitude of binding energy and the results were visualized by PyMOL 2.6.0.

### Statistical analysis

All data are presented as the means ± SEM and analyzed by GraphPad Prism 9 software. Comparisons between groups were performed via unpaired *t* tests, and data with unequal variances were compared with the Mann‒Whitney U test. Normally distributed data from multiple groups were compared with ANOVA. *P* < 0.05 was considered statistically significant.

## Results

### Macrophage polarization and the MIF-CD74-NF-κB cascade are involved in CTX- induced ovarian fibrosis

**Fig. 1:**
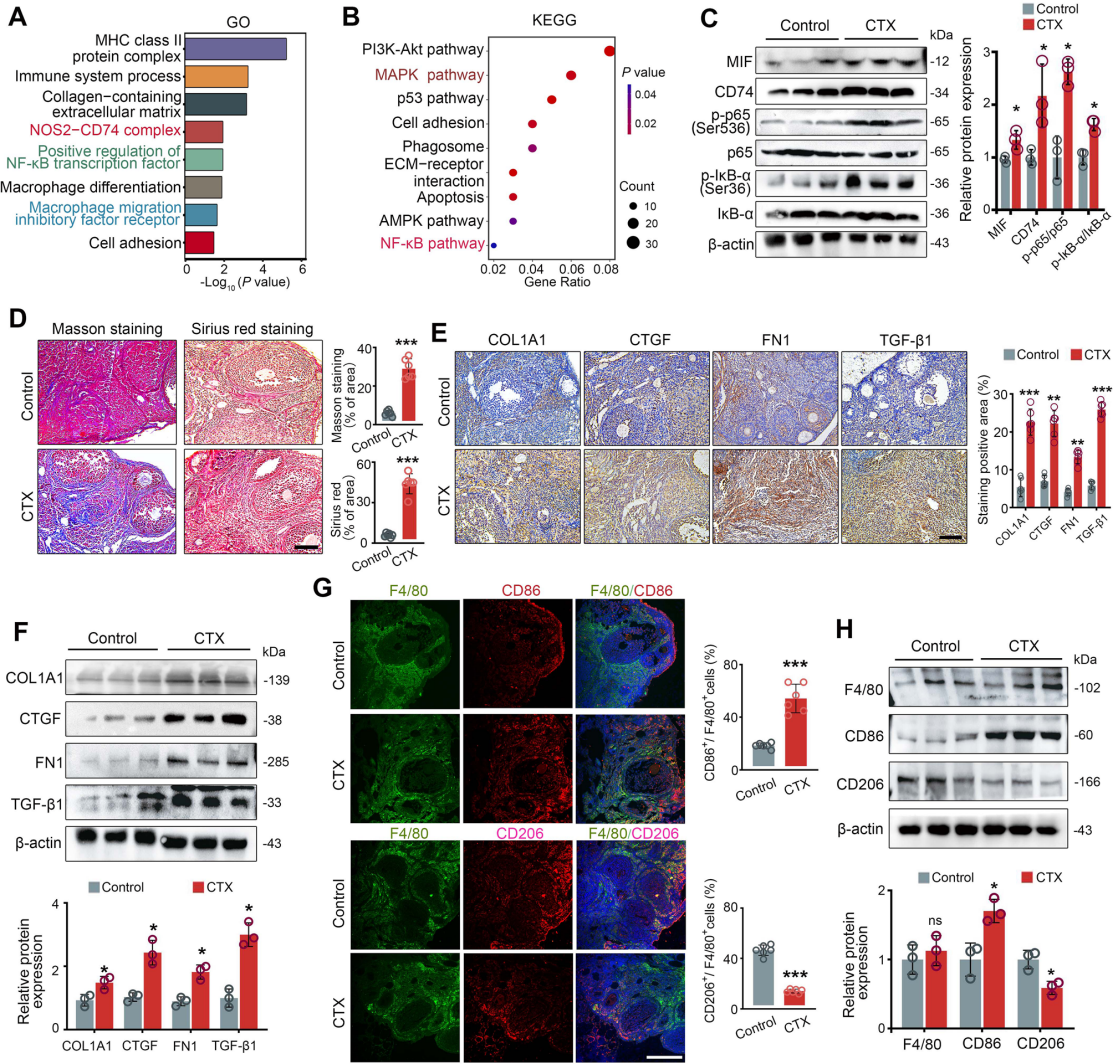
Activation of the MIF-CD74-NF-κB cascade and fibrosis in CTX-induced POF. (**A**) Go terms and (**B**) KEGG pathway enrichment analysis for DEGs in ovaries of CTX-treated versus control ovaries (*n* = 3 mice per group). (**C**) Western blot analysis and quantification of MIF, CD74, p-p65 and p-IκBα levels. (**D**) Fibrosis assessment by Masson trichrome blue and Sirius red red staining. (**E** and **F**) Immunostaining and western blot of ECM-related proteins (FN1, COL1A1, CTGF, and TGF-β1). (G and H) Macrophage markers (F4/80, CD86 and CD206) levels were analyzed by immunofluorescence and western blot. Scale bars: 100 μm. *P < 0.05, **P < 0.01 and ***P < 0.001 for the indicated comparisons

After 14 days of CTX treatment, bulk RNA-seq was used to examine DEGs in the ovaries of female C57BL/6J mice treated with and without CTX. In total, 442 up- and 378 down- regulated differentially expressed gene (DEGs) were extracted (Additional file: Figure. S1). GO enrichment analysis highlighted NOS2-CD74 complex, positive regulation of NF-κB transcription factor, macrophage migration inhibitory factor receptor (Fig. 1A), while KEGG pathways included PI3K-Akt, MAPK and NF-κB signaling pathways (Fig. 1B). To validate these findings, western blot analysis demonstrated increased expression of MIF, CD74, p-p65 and p-IκB-α in CTX-treated ovaries, indicating activation of both MIF-CD74 and NF-κB pathways, which have been shown to be involved in macrophage polarization [22, 23] (Fig. 1C). Masson and Sirius red staining showed elevated fibrotic areas and collagen deposition in CTX-induced ovaries (Fig. 1D); further supported by upregulated expression of ECM-related genes (COL1A1, CTGF and FN1) and TGF-β1 proteins via immunostaining and western blot (Fig. 1E and F). These findings support the hypothesis that TGF-β1 was activated in the ovaries of mice in the CTX-induced POF group. In particular, while the M1 macrophage marker CD86 increased and M2 marker CD206 decreased, F4/80 expression remained unchanged, suggesting altered macrophage polarization without total macrophage infiltration in CTX-induced ovaries (Fig. 1G and H).

### Metformin improves follicle preservation and hormone regulation in CTX-induced POF

**Fig. 2:**
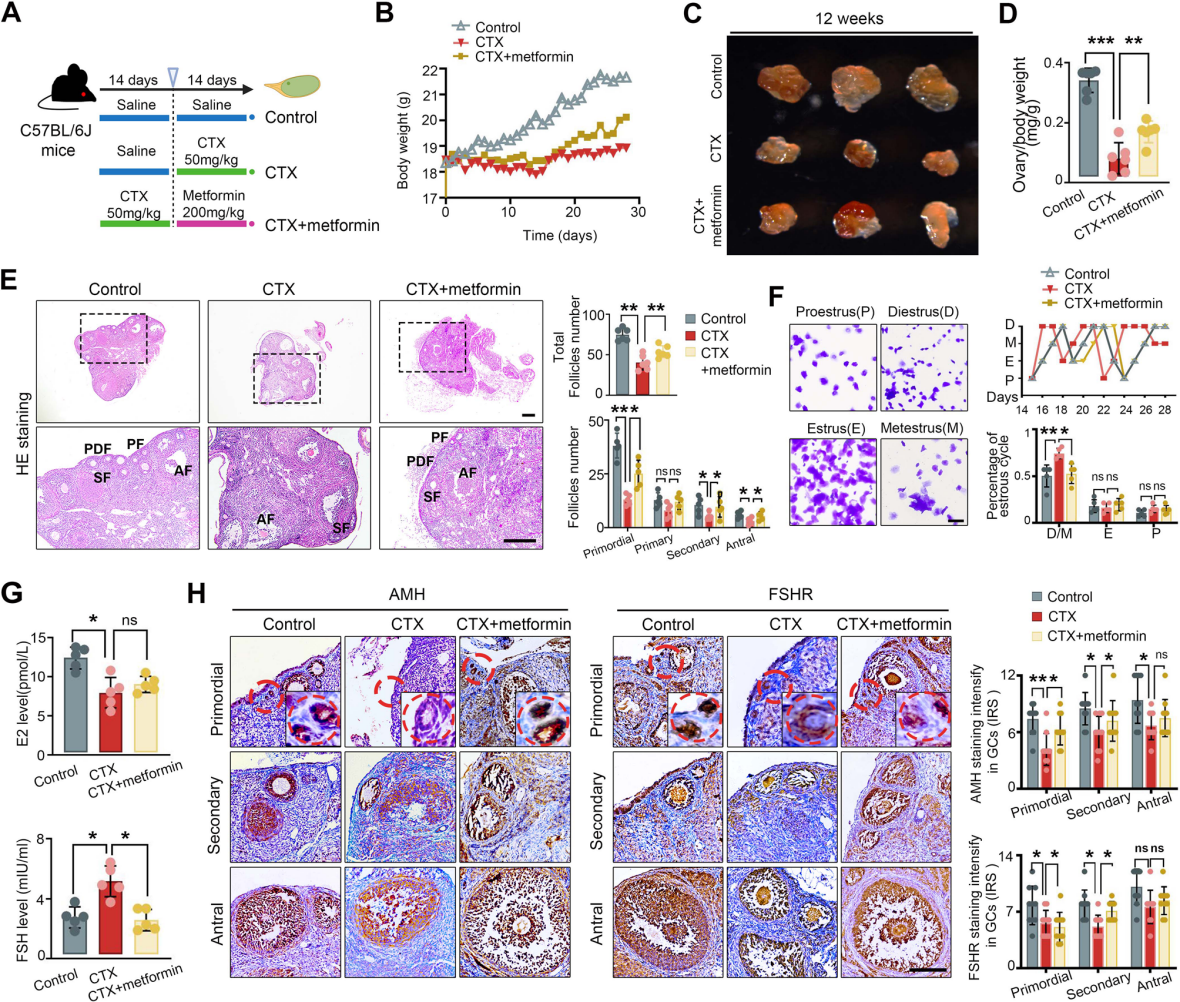
Effects of metformin on folliculogenesis and hormone levels in CTX- induced POF**.** (**A**) Schematic diagram of CTX and/or metformin treatment in mice, showing the experimental groups and treatment timeline. (**B**) Average mouse weight throughout the injection period. (**C**) Representative gross images of ovaries and (**D**) the ovary/body weight ratio in mice. (**E**) Representative histology images of ovary sections from tumor-bearing mice and bar chart showing the quantification of total, primordial, primary, secondary, and antral follicles in mice. (**F**) Representative estrous cycles of mice over 28 consecutive days and quantitative analysis of the estrous cycles. (**G**) Serum E2 and FSH levels in mice were measured by ELISA. (**H**) Immunostaining for AMH and FSHR expression in GCs in follicles at different developmental stages and quantification of the percentages of positive GCs per follicle within the ovaries of each group. PDF, primordial follicle; PF, primary follicle; SF, secondary follicle; AF, antral follicle. AMH, anti-Mullerian hormone; FSHR, follicle-stimulating hormone receptor. Scale bars: 200 μm. *P < 0.05, **P < 0.01, ***P < 0.001 and ****P < 0.0001 for the indicated comparisons

To investigate whether metformin improves ovarian function in CTX-induced POF, we assessed parameters related to follicle development, hormone levels, and ovarian morphology. A previous study demonstrated that a 3-week lavage of metformin 300 mg/kg improved ovarian function in CTX-induced POF mice [8]. However, intraperitoneal (i.p.) administration of metformin had a higher absolute uptake in the ovary compared with oral or gavage routes of administration [24] and 200 mg/kg was the recommended dose in mice [25] (Fig. 2A). The body weight of CTX-treated mice was significantly lower than that of control mice (*P* < 0.0001), while metformin administration partially ameliorated this decrease (Fig. 2B). Ovary size was significantly reduced in the CTX group compared to controls (*P* = 0.0017), but metformin treatment led to a significant increase in ovary size (Fig. 2C and D). The ovarian index was significantly higher in the metformin group than in the CTX group (*P* = 0.003) (Fig. 2D). Moreover, CTX treatment significantly decreased the total number of follicles, particularly primordial and secondary follicles, compared to controls (Fig. 2E). These data suggest that metformin improved follicle loss induced by CTX.

The estrous cycle, confirmed by vaginal smears, showed that CTX-treated mice exhibited prolonged diestrus stages, which was partially ameliorated by metformin treatment, as revealed by a microscopic analysis of quantitative analysis of cell populations (Fig. 2F). Accordingly, in CTX-treated mice, the serum E_2_ levels were significantly lower in CTX-treated mice compared to controls (11.803 ± 1.392 vs. 7.974 ± 1.967, *P* = 0.015), while the FSH levels of the CTX group were significantly higher (2.750 ± 0.706 vs. 5.166 ± 1.042, *P* = 0.022), thus indicating POI in the CTX-treated mice. Metformin administration reduced FSH levels (*P* = 0.013) and trended towards increased E_2_ levels (*P* = 0.0648) (Fig. 2G). In addition, the expression of anti-Mullerian hormone (AMH) and follicle stimulating hormone receptor (FSHR) was detected by immunohistochemical analysis. It showed that the expression of AMH and FSHR was decreased in the GCs of CTX-induced ovaries compared to the control. In contrast, the majority of follicles were observed in the ovaries from the metformin group, with a significantly increased secretion of AMH and FSHR (Fig. 2H). These data demonstrated metformin improved hormone levels altered by CTX.

### Metformin reduces ovarian fibrosis and inhibits MAPK/JNK pathway

**Fig. 3:**
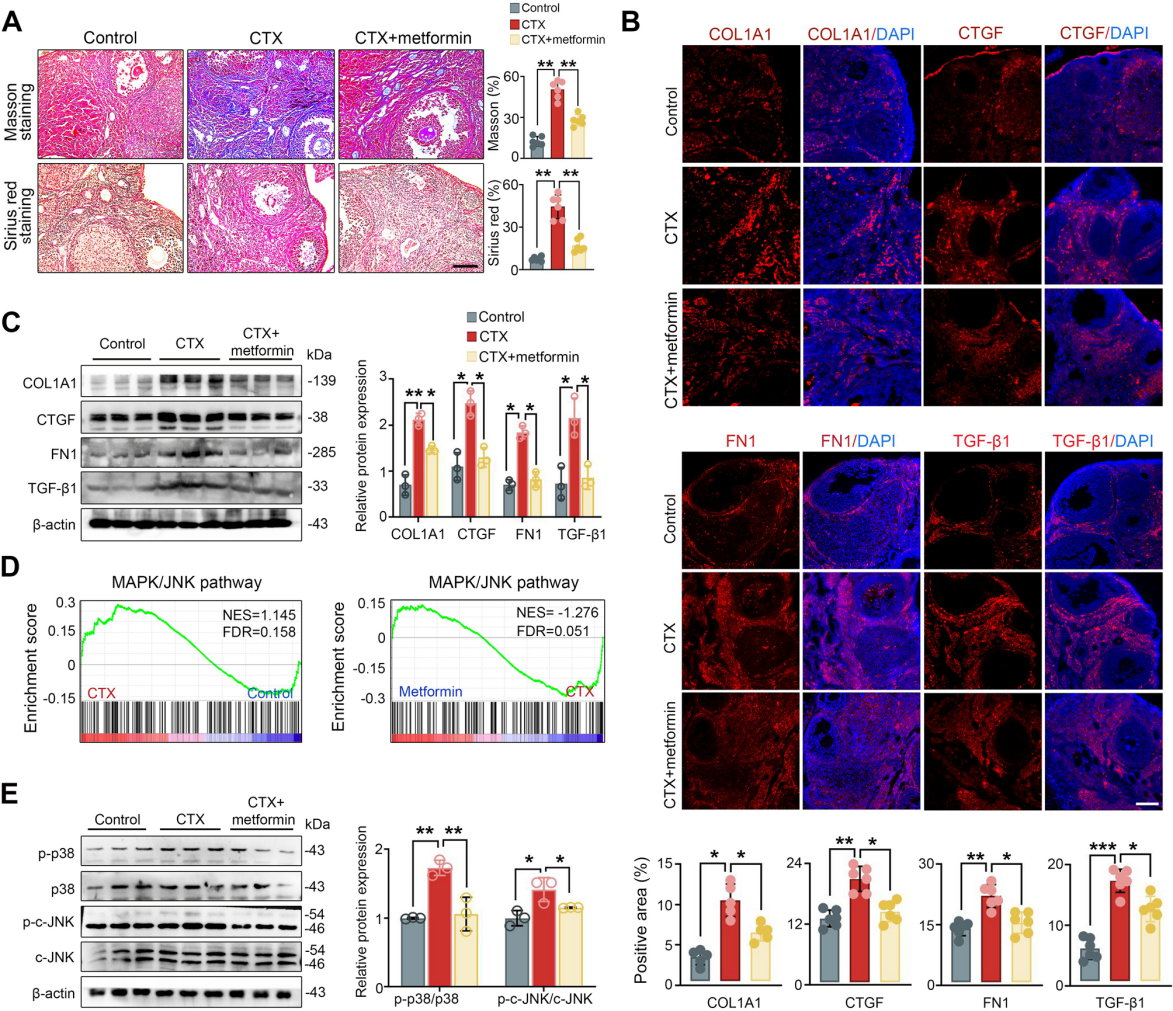
Reduced ovarian fibrosis and inhibition of the MAPK/JNK pathway after metformin treatment**.** (**A**) Masson trichrome and Sirius Red staining of ovarian tissue. (**B**) Immunofluorescence staining for COL1A1, CTGF, FN1 and TGF-β1 expression. (**C**) Western blot analysis of ECM-related proteins expression. (**D**) MAPK/JNK pathway is enriched in CTX-treated and metformin-treated ovaries. (**E**) Western blot analysis of proteins involved in the MAPK/JNK pathway. Scale bars: 100 μm. *P < 0.05, **P < 0.01, ***P < 0.001 and ****P < 0.0001 for the indicated comparisons

Masson and Sirius red staining revealed increased fibrotic areas and collagen deposition in CTX-induced POF ovaries (Fig. 3A); these results were further confirmed by immunostaining analysis showing elevated expression of COL1A1, CTGF, FN1, and TGF-β1 proteins (Fig. 3B). These findings suggest that ECM-related protein expression was elevated in the ovaries of mice in the CTX-induced group. In contrast, metformin treatment reversed reduced CTX-induced ovarian fibrosis, as demonstrated by Masson and Sirius red staining, and the reduced expression of COL1A1, CTGF, FN1 and TGF-β1 expression in ovarian stroma (Fig. 3A and B). Western blot analysis also demonstrated that metformin reversed the CTX-induced upregulation of ECM-related protein levels (Fig. 3C).

Furthermore, the MAPK signaling cascade, particularly c-Jun N-terminal kinase (JNK) JNK branch, emerges as a pivotal mediator of TGF-β signaling [13]. GSEA enrichment analysis of biological processes demonstrated MAPK/JNK was activated in CTX-treated ovaries while it was inhibited in metformin-treated ovaries (Fig. 3D and Additional file: Figure S1 and S2). Western blot analysis also demonstrated that metformin reversed the CTX-induced upregulation of c-JNK and p38MAPK protein levels (Fig. 3E). These data suggest that metformin exerts protective effects by attenuating the fibrotic process involving the MAPK/JNK signaling pathway.

### Metformin reduced the M1/M2 macrophage ratio and suppressed the MIF-CD74-NF-κB cascade in POF

**Fig. 4:**
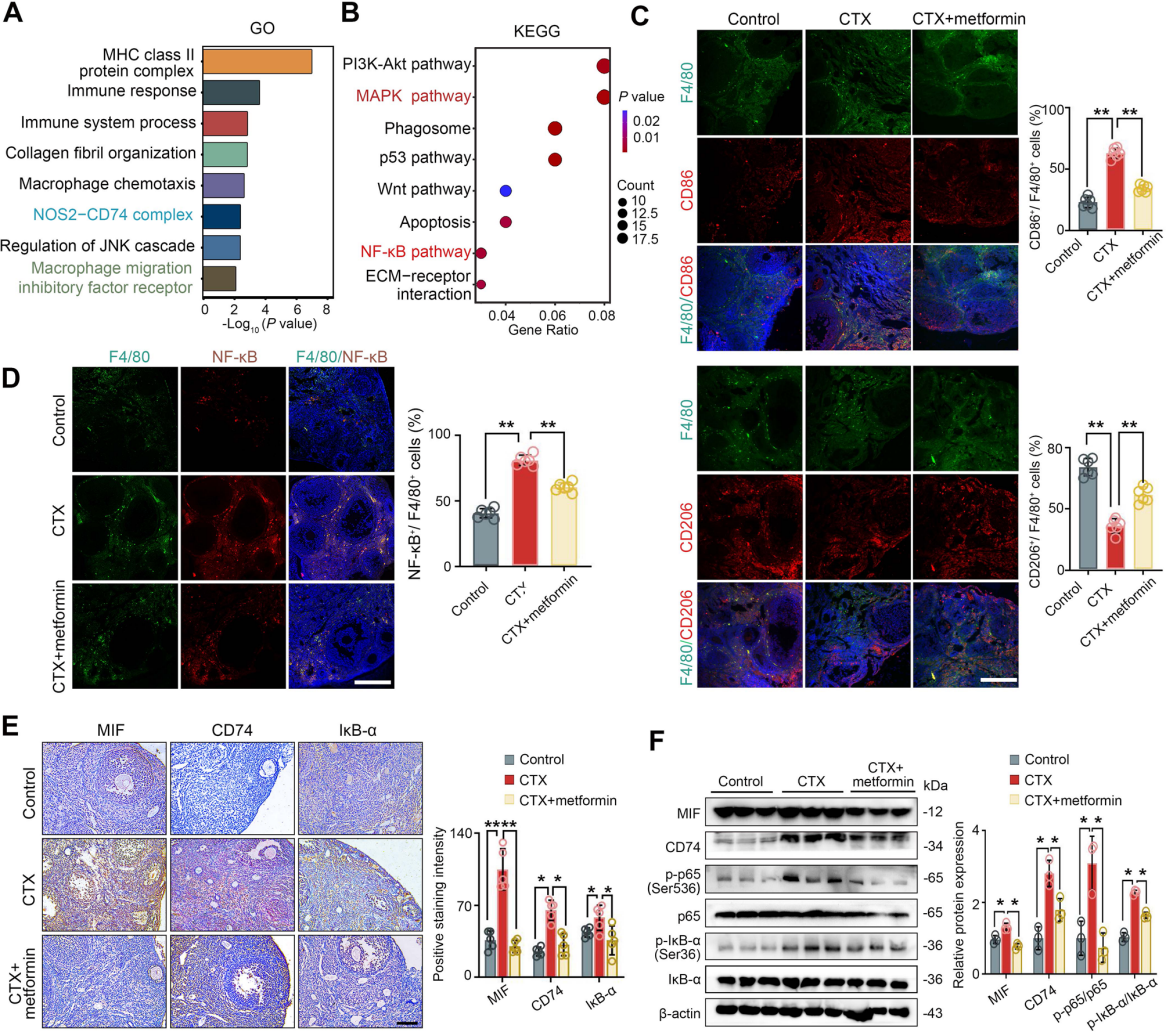
Reduced M1/M2 macrophage ratio and suppression of the MIF-CD74-NF-κB cascade after metformin treatment**.** (**A**) Go terms and (**B**) KEGG pathway enrichment analysis for DEGs in ovaries of CTX alone or CTX + metformin -treated ovaries (*n* = 3 mice per group). (**C**) Immunofluorescence staining for macrophage (F4/80), M1 (CD86/F4/80), and M2 (CD206/F4/80) infiltration in ovary. (**D**) Immunofluorescence staining for NF-κB and macrophage expression. (**E**) Immunostaining for MIF, CD74 and IκB-α expression. (**F**) Western blot analysis of MIF-CD74-NF-κB pathway proteins. Scale bars: 100 μm. *P < 0.05, **P < 0.01, ***P < 0.001 and ****P < 0.0001 for the indicated comparisons

RNA-seq analysis was used to explore the differences in gene expression between the ovaries of mice treated with CTX alone and those treated with CTX + metformin (Additional file: Figure S2). In the metformin-treated ovaries, GO enrichment analysis highlighted immune system, MHC class protein complex, MIF-related pathways (Fig. 4A), while KEGG pathways included PI3K-Akt, MAPK and NF-κB signaling pathway (Fig. 4B), suggesting that metformin may function through the MIF-CD74 pathway. The immunofluorescence results showed that metformin administration restored while CTX induced an increase in the infiltration of M1-type macrophages (F4/80^+^CD86^+^) and a decrease in the infiltration of M2-type macrophages (F4/80^+^ CD206^+^) (Fig. 4C). NF-κB serves as a key transcription factor determine polarization of M1 and M2 [26]. Further analysis of NF-κB pathway proteins expression demonstrated that metformin reversed CTX-induced expression changes of NF-κB in F4/80^+^ macrophages (Fig. 4D), as well as IκB-α, MIF and CD74 in ovary of mice (Fig. 4E), as indicated by immunofluorescence and western blot analysis (Fig. 4F). Western blot analysis also demonstrated reduced the phosphorylation of the activation of p-IκB-α and p-p65 proteins compared with that in the CTX group (Fig. 4F).

### Metformin modulates macrophage polarization through the MIF-CD74 axis

**Fig. 5:**
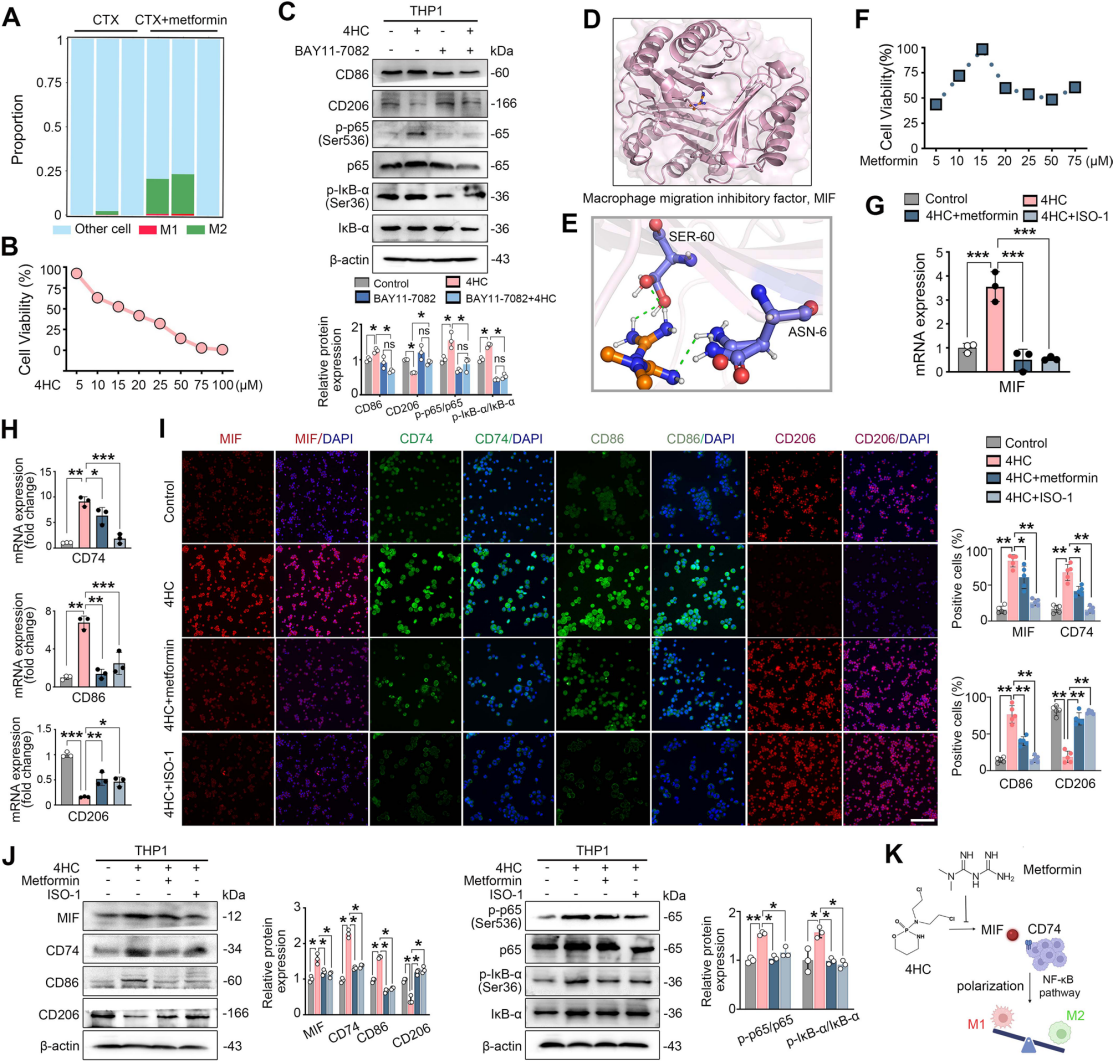
Metformin modulated macrophage polarization is mediated by the MIF-CD74 axis**.** (**A**) Proportions of M1 and M2 macrophage subpopulations infiltrating ovaries from the CTX-treated and CTX + metformin-treated groups. (**B**) Dose–response curve estimating IC50 values of 4HC or metformin in THP-1 cells after 24 h. (**C**) Cells treated with the NF-κB inhibitor BAY11-7082 with or without 4HC induction. The expression of macrophage markers and NF-κB key proteins were detected by western blot. (**D**) 3D structure of MIF. (**E**) The possible molecular docking results between metformin and MIF were obtained from the SwissDock online server. (**F**) Dose–response curve estimating IC50 values of metformin in THP-1 cells after 24 h. (**G**) qPCR analysis of MIF mRNA in THP-1 cells treated with 4HC alone or in combination with metformin or ISO-1. (**H**) mRNA expression levels of CD74, CD86 and CD206 as detected by RT-qPCR. (**I**) Immunofluorescence staining for MIF, CD74, CD86 and CD206. (**J**) Western blot analysis of proteins involved in the MIF/CD74/NF-κB pathway. (**K**) Diagram describing the associated MIF/CD74/NF-κB pathway in the metformin triggered macrophage homeostasis in the imbalance of macrophage polarization induced by CTX. Scale bars: 300 μm. *P < 0.05, **P < 0.01, ***P < 0.001 and ****P < 0.0001 for the indicated comparisons

Using the CIBERSORT algorithm, a difference in macrophage infiltration was identified between ovaries treated with CTX and those treated with CTX combined metformin. The metformin-treated group showed a greater proportion of M2 macrophages (0.6% vs. 14%, *P* < 0.01), but no significant difference in the proportion of M1 macrophages (0.08% vs. 0.2%, *P* = 0.1716) (Fig. 5A). Our previous work demonstrated that CTX has a role in macrophage polarization in vitro [7], and IC50 value of 4HC was 15 µM in THP-1 cells at 24 h (Fig. 5B). The NF-κB inhibitor BAY11-7082 was used alone or together with 4HC in THP1 cell. 10 µM BAY11-7082 enhanced CD206 expression and simultaneously reduced p-IκB-α, p-p65 and CD86 expression, suggesting that 4HC could activate NF-κB pathway and enhance M1/M2 macrophage ratio in THP1 cell (Fig. 5C).

To validate the regulatory role of metformin in macrophages, the targets of metformin were predicted by PubChem databases, and MIF was obtained (Fig. 5D). Molecular docking results were obtained from the SwissDock online server by uploading the gene information of MIF and metformin. Metformin is predicted to bind directly to the MIF protein, where residue Cys59 forms a hydrogen bonding interaction and residue Asn97 forms a hydrocarbon interaction with the compound (Fig. 5E).

In THP-1 cells, the IC50 value of metformin was 25 µM at 24 h (Fig. 5F). Since previous studies reported that the resting state of macrophages is not affected by ISO-1 (MIF inhibitor) [27], THP-1 cells were treated with 4HC alone or in combination with ISO-1. As expected, RT-qPCR analysis revealed that MIF mRNA expression was increased by 255% following 4HC treatment, and this upregulation was reversed by either metformin or ISO-1 (Fig. 5G). Moreover, both metformin and ISO-1 reversed the 4HC-induced increase in CD86 and CD74, and decrease in CD206 at both the mRNA and protein level as confirmed by RT-qPCR and immunofluorescence assays (Fig. 5H and I).

Furthermore, western blot analyses showed that the 4HC treatment elevated the expression of MIF, CD74, p-IκB-α and p-p65 proteins. This effect was abolished by metformin or MIF inhibition via ISO-1, which upregulates CD86 and MIF/CD74 proteins and downregulates CD206 in 4HC-induced THP-1 cells (Fig. 5J). Moreover, our results showed there was no significant decrease in the total number of macrophages in the CTX group compared with the control group (448 ± 28 cells/mm² vs. 403 ± 15 cells/mm²; *P* = 0.2273). This supports the idea that CTX may have no obvious effect on macrophage recruitment. In vitro, light and crystal violet images showed no significant difference in THP-1 cell number in the ISO-1 group compared with the control group (315,443 ± 5,389 cells vs. 303,214 ± 7,730 cells/mm²; *P* = 0.2641) (Reply Fig. 2B). Fluorescence analysis showed decreased MIF expression and increased CD206 expression but no change on F4/80 expression after ISO-1 treatment (Reply Fig. 2C). MIF/CD74 signaling may contribute to macrophage polarization but not recruitment (Additional file: Figure S3). Collectively, these findings suggest that both metformin and MIF inhibitor ISO-1 modulate M1 and M2 macrophage polarization, likely though the MIF/CD74 signaling pathway (Fig. 5K).

### Co-culture with macrophages promotes the fibrotic response of fibroblasts through the MAPK/JNK pathway

**Fig. 6:**
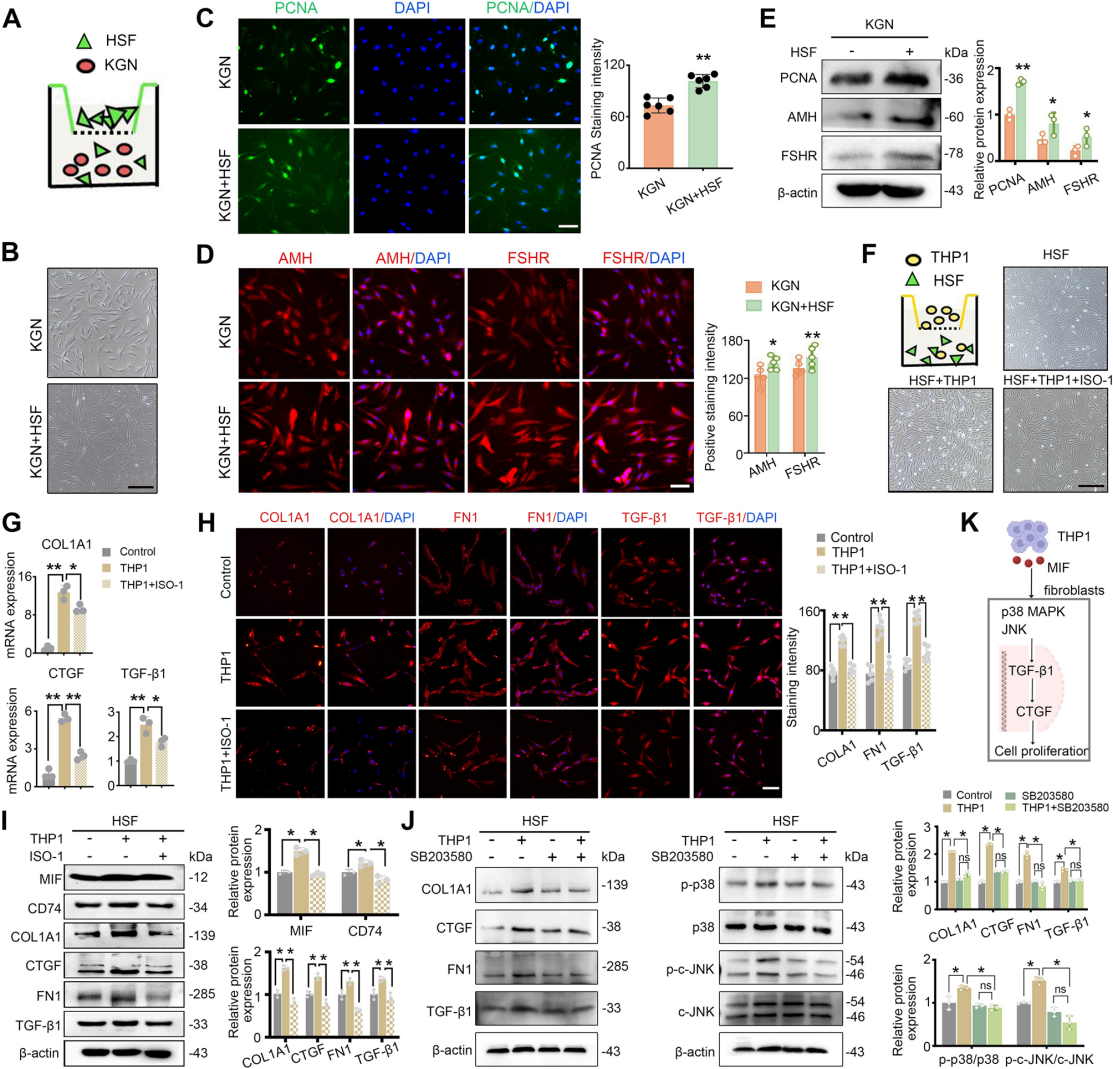
Co-culture with fibroblasts or macrophages modulates granulosa cell function or fibrotic response. (**A**) Schematic of KGN-HSF cells co-culture. (**B**) Morphology of KGN cells co-cultured or not co-cultured with HSF cells. (**C**) Immunofluorescence staining for PCNA and (**D**) AMH and FSHR levels. (**E**) Western blot analysis for PCNA, AMH and FSHR proteins. (**F**) Schematic of HSF-THP1 co-culture. (**G**) HSF morphology under indicated conditions. (**H**-**J**) ECM-related gene/proteins analyzed by RT-qPCR, immunofluorescence, and western blot. (**K**) Cells were treated with p38MAPK inhibitor under HSF-THP1 co-culture. The expression level s of ECM-related and MAPK/JNK pathway key proteins were detected by western blot. (**L**) Diagram describing the mechanisms of macrophage-derived MIF may trigger fibrotic response in fibroblasts. Scale bars: 100 μm in C, D and I, and 300 μm in B and G. *P < 0.05, **P < 0.01, ***P < 0.001 and ****P < 0.0001 for the indicated comparisons

Ovarian fibrosis is caused by activation of fibroblasts and ECM deposition in the ovary [28]. To investigate fibroblast-macrophage crosstalk in ovarian fibrosis, we first established a Transwell co-culture system. Since isolating ovarian-derived fibroblasts risks tumor cell contamination, and human skin fibroblasts (HSF) have been shown to functionally resemble ovarian fibroblasts in supporting follicular development [29, 30], we thus used HSF as a surrogate model. A non-contact co-culture model using a 0.4 μm Transwell system, with KGN cells seeded in the lower chamber and HSF cells in the upper chamber, was established to mimic paracrine interactions between GCs and fibroblasts (Fig. 6A). The presence of HSF cells caused distinctive changes in cell morphology and increases in growth in KGN cells (Fig. 6B). Immunofluorescence assays demonstrated that the intensity of nuclear PCNA staining was significantly increased in KGN cells co-cultured with HSF cells compared with KGN cells cultured alone following treatment for 24 h, as determined by western blot analysis (Fig. 6C and E). To establish whether the expression levels of growth factors could relate to GC function, the expression of AMH and FSHR were examined by immunofluorescence and western blot assays (Figure. 6D and E). Together, co-culture with HSF promoted KGN cell proliferation evidenced by unregulated PCNA and enhanced GC functional markers AMH and FSHR, suggesting a supportive role of fibroblasts in GC activity.

Next, to investigate whether macrophages could further modulate HSF fibrotic activity and the role macrophage-derived MIF, ECM-related genes were evaluated in HSF cells co-culture with THP1 cells and/or treated with ISO-1 (Fig. 6F). The presence of THP1 cells caused morphological changes, increased COL1A1, CTGF and TGF-β1 at both the mRNA and protein level. Notably, MIF inhibition by ISO-1 reversed these effects (Fig. 6G-J), implicating macrophage-derived MIF as a key mediator of the HSF fibrotic response.

Furthermore, the p38MAPK inhibitor SB203580 was used in the HSF-THP1 co-culture model. 10 µM SB203580 reduced COL1A1, CTGF, FN1 and TGF-β1, p-JNK and p-p38 expression (Fig. 6K). These data suggested that THP1 could activate MAPK/JNK signaling in HSF cells.

### CD74 promotes the fibrotic response of fibroblasts through the MAPK/JNK pathway

**Fig. 7:**
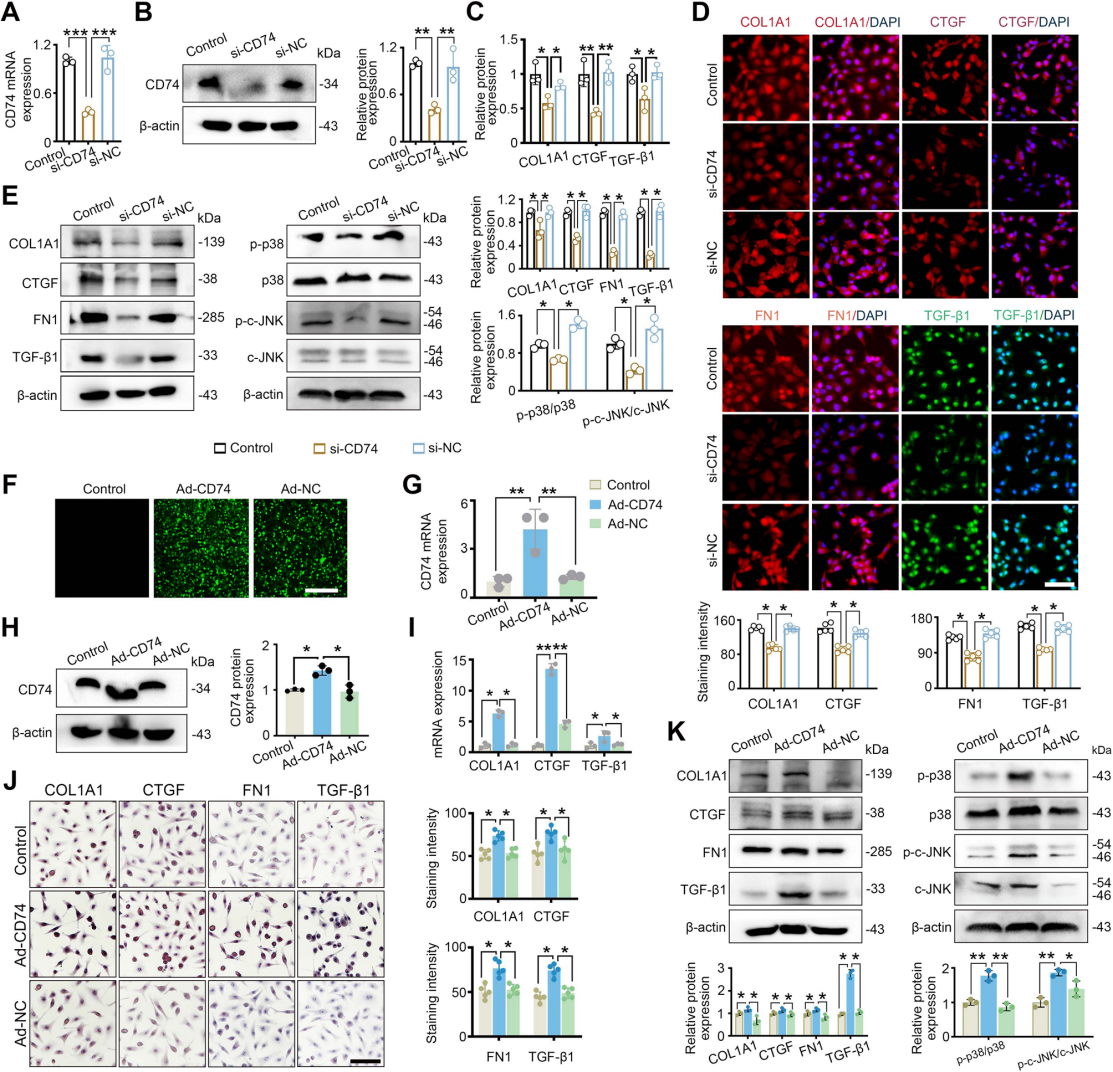
CD74 promotes fibrotic response of fibroblast through the MAPK/JNK pathway**.** (**A**) qPCR and (**B**) western blot analysis were used to measure the transfection efficiency and CD74 expression in si-CD74 in HSF cells. (**C**-**E**) ECM-related gene/proteins analyzed by RT-qPCR, immunofluorescence, and western blot. (**F**) Immunofluorescence staining showing mNeonGreen expression in transfected HSF cells. (**G**) CD74 mRNA and (**H**) protein expression was determined by RT-qPCR and western blot analysis, respectively. (**I**-**K**) ECM-related gene/proteins analyzed by RT-qPCR, immunostaining and western blot, and MAPK/JNK pathway key proteins were detected by western blot. Scale bars: 100 μm in D and J, and 200 μm in F. *P < 0.05 and **P < 0.01

To determine a direct effect of CD74 on fibroblasts, CD74 was knocked down by siRNA. RT-qPCR and western blot analysis confirmed CD74 was reduced in response to siRNA (Fig. 7A and B). Knockdown of CD74 downregulated COL1A1, CTGF and TGF-β1 mRNA and protein expression, as determined by qRT-PCR, immunofluorescence and western blot analysis in HSF (Fig. 7C-E). Western blot analysis also demonstrated that knockdown of CD74 downregulated c-JNK and p38MAPK protein levels (Fig. 7E).

Meanwhile, a CD74 overexpression plasmid was constructed, and HSF cells were transfected with the plasmid Ad − CD74 (Ad-CD74) (Additional file: Figure S4). There was a significantly higher expression of CD74 in Ad-CD74 cells compared to empty vector (Ad-NC), as evaluated by qRT-PCR, green fluorescence and western blot analysis (Fig. 7F-H). Ad-CD74 cells demonstrated an increase of TGF-β1, COL1A1 and CTGF mRNA and protein expression, as determined by qRT-PCR, immunohistochemical staining and western blot analysis. Furthermore, Ad-CD74 cells demonstrated an increase in phosphorylation of p38MAPK and c-JNK (Fig. 7H -J).

## Discussion

Ovarian fibrosis is a hallmark of pathological conditions mostly linked to conditions like POF, age-related ovarian dysfunction, and polycystic ovary syndrome (PCOS). Previous studies have shown that metformin mitigates ovarian fibrosis though immunomodulation of profibrotic and inflammatory signaling pathways in aged or high-fat diet murine models [9, 18, 19]. However, the antifibrotic effect of metformin and the role of MIF/CD74 signaling pathway have not been studied in ovarian fibrosis induced by CTX. The present study demonstrated that CTX-induced ovarian fibrosis in mice is mechanistically associated with activation of the MIF/CD74 pathway. In vitro experiments revealed that CTX significantly increased the CD86^+^ (M1 marker)/CD206^+^ (M2 marker) macrophage ratio while upregulating MIF/CD74 signaling. Importantly, metformin administration or pharmacological inhibition of MIF effectively restored macrophage homeostasis by suppressing NF-κB activation, which could in turn lead to ECM production in fibroblasts. Furthermore, CD74 knockdown and overexpression models demonstrated that this receptor regulates fibrotic responses through MAPK/JNK pathway modulation in fibroblasts. This finding suggests that the MIF/CD74 signaling pathway may be a previously unreported therapeutic target for chemotherapy-induced ovarian damage, and revealed a macrophage-mediated mechanism by which metformin exerts its fertility-preserving effects during CTX treatment, supporting it could be a promising novel fertility preserving agent during chemotherapy.

Macrophages exhibit plasticity between pro-inflammatory M1 and anti-inflammatory M2 phenotypes, where excessive M2 dominance over M1 macrophages may favor pathological fibrosis in multiple organs [26, 31]. While metformin has demonstrated therapeutic potential in modulating the inflammatory microenvironment, its effects on macrophage polarization in ovarian fibrosis remains controversial. For instance, conflicting studies report that metformin may either increase or decrease the M1/M2 macrophage ratio in different pathological contexts [9, 28]. In age-related ovarian fibrosis, where M2 macrophages predominate and exacerbate fibrotic progression, metformin treatment effectively elevates the M1/M2 macrophage ratio to restore tissue homeostasis and mitigate fibrosis [9]. This contrasts with CTX-induced ovarian damage, where CTX activates M1 macrophage polarization both in vitro and in vivo [7], as well as excessive M1 polarization in PCOS models [7, 28, 32]. Moreover, metformin can lessen fibrosis in ovaries of postmenopausal women [9], decreases pro-inflammatory factors secreted by M1 macrophages and reduces collagen deposition in ovaries of POF or obese mice [8, 28]. Consistent with these reports, our results demonstrated metformin alleviates CTX-induced ovarian fibrosis through bidirectional regulation of macrophage plasticity: it suppresses excessive M1 polarization in vitro and in CTX-treated ovaries while concurrently attenuating M2-driven fibroblast proliferation. This dual-phase mechanism aligns with metformin’s reported ability to preferentially inhibit M1 macrophages while promoting M2 polarization in tumor microenvironments [33], suggesting a context-dependent therapeutic strategy.

MIF has been suggested to be a key mediator that promotes fibrosis by activating CD74 in liver, renal and pulmonary fibrosis models [10, 11, 34]. Notably, while MIF is also elevated in ovarian tissues and serum from PCOS subjects [35], its specific role through the CD74 pathway remains unexplored in POF pathogenesis. The NF-κB pathway, a well-established central regulator of macrophage polarization, exhibits dual functionality. Its activation induces pro-inflammatory M1 polarization while suppressing anti-inflammatory M2 macrophage development [36]. MIF demonstrates parallel effects by promoting M1 polarization and inhibiting M2 polarization [22], with mechanistic studies revealing that MIF deficiency impairs NF-κB activation and attenuates M1 responses [23]. This functional synergy is further substantiated by the finding that MIF binding to CD74 on macrophages triggers NF-κB activation, thereby driving pro-inflammatory M1 polarization [37]. Thus, these findings suggest an intricate interplay between the MIF/CD74 axis and NF-κB signaling in modulating macrophage phenotypic switching. The present study identified MIF/CD74 axis as a key pivotal regulator in CTX-induced macrophage polarization. Enhanced phosphorylation of p65 and total p65 accumulation in CTX-treated ovaries showed correlation with decreased CD86 and increased CD206 expression, indicating NF-κB pathway activation determines macrophage polarization balance in POF. Pharmacological interventions including metformin, MIF inhibitors, and NF-κB inhibitors consistently reduced M1-related gene expression while augmenting M2 markers. These data strongly support that metformin facilitates M1-to-M2 macrophage repolarization primarily through blockade of the MIF/CD74-induced NF-κB activation cascade.

Fibroblasts constitute the primary effector cells in fibrotic response. Although CTX-induced fibrosis has been well characterized, the precise mechanisms underlying macrophage-fibroblast interactions and metformin’s regulatory effects on ovarian stromal remodeling remain elusive. TGF − β1, a master regulator of ECM deposition, exerts its pro-fibrotic effects through coordinated regulation of collagens, fibronectin (FN) and connective tissue growth factor (CTGF) expression in the ovary [13, 38]. Mechanistically, c- JNK activation facilitates TGF-β receptor substrate phosphorylation, thereby amplifying downstream fibrogenic signals [39]. This pathogenic axis has been mechanistically linked to excessive follicular atresia and stromal fibrosis in both PCOS and POF models [38, 40, 41]. Notably, pharmacological inhibition of JNK signaling has shown therapeutic efficacy against ovarian fibrosis in PCOS models [41], suggesting pathway-specific targeting potential. In vitro studies demonstrate that MAPK/JNK activation not only initiates fibroblast transdifferentiation but also synergizes with TGF-β1 to upregulate pro-fibrotic gene networks [42, 43]. Additionally, the interaction between MIF and CD74 augments TGF-β1 production in fibroblasts by via concurrent activation of MAPK and JNK pathway [12, 44].

The present work demonstrated metformin or MIF inhibitor not only promotes macrophage polarization from M1 to M2 phenotype but also suppresses fibroblasts response in CTX-induced mice and in co-culture systems. This suppression is evidenced by reduced expression of fibrogenic mediators, including COL1A1, CTGF, and FN1. RNA-seq analysis analysis further revealed that CTX treatment significantly upregulates genes associated with the MAPK pathway, particularly those involved in p38MAPK and JNK signaling in ovarian tissues. Importantly, macrophages co-cultured with fibroblasts markedly increased phosphorylation levels of MAPK/JNK pathway proteins, including p-p38 MAPK, c-JNK compared to mono-cultured fibroblasts. These findings suggest that macrophage-derived paracrine signals critically promote fibroblast fibrogenesis through activation of the MAPK/JNK pathway, thereby enhancing ECM deposition and ovarian fibrosis. Furthermore, functional studies demonstrated that CD74 knockdown attenuated ECM-related genes expression in fibroblasts, whereas CD74 overexpression exacerbated it. Collectively, our data indicate that the anti-fibrotic effects of metformin are mediated via suppression of the MIF/CD74 signaling axis, which modulates both macrophage polarization and MAPK/JNK-driven fibroblast activation.

## Conclusion

In summary, the present study identified a mechanism by which metformin ameliorates CTX-induced ovarian fibrosis through modulation of the MIF/CD74 signaling axis. Metformin not only promotes macrophage phenotypic switching from M1 to M2 through NF-κB pathway inhibition, but also suppresses macrophage-derived fibroblast responsiveness to TGF-β1 via CD74-mediated downregulation of MAPK/JNK signaling. These findings extend our understanding beyond metformin’s established role in improving GC function, revealing its capacity to regulate fibrosis by targeting macrophage-fibroblast crosstalk. Clinically, metformin administration during chemotherapy could preserve ovarian reserve not only by protecting follicular components but also by preventing ECM remodeling. The reduction of fibrosis in response to metformin could have significant implications for improving ovarian health in patients undergoing chemotherapy or suffering from other ovarian pathologies associated with fibrosis. Further research is needed to confirm these findings and explore the broader implications of metformin in the treatment of fibrotic diseases.

## Supplementary Information

Below is the link to the electronic supplementary material.

Supplementary Material 1

Supplementary Material 2

Supplementary Material 3

Supplementary Material 4

Supplementary Material 5

## Data Availability

The datasets generated and used in this study are available from the corresponding author on reasonable request. The original images supporting the conclusions of this article are publicly accessible on Figshare under the 10.6084/m9.figshare.29964953.

## Abbreviations

CTX: Cyclophosphamide
POF: Premature ovarian failure
ECM: Extracellular matrix
MIF: Macrophage migration inhibitory factor
TGF-β1: Transforming growth factor-β1
NF-κB: NF-nuclear factor kappa B
RNA-seq: RNA sequencing
4HC: 4-hydroperoxycyclophosphamide
DEGs: Differentially expressed genes
KEGG: Kyoto Encyclopedia of Genes and Genome
NCBI: National Center for Biotechnology Information
ELISA: Enzyme-linked immunosorbent assay
FSH: Follicle-stimulating hormone
E2: Estradiol
H&E: Hematoxylin and eosin
HSF: Human skin fibroblasts
siRNA: Small interfering RNA
i.p.: Intraperitoneal
AMH: Anti-Mullerian hormone
FSHR: Follicle stimulating hormone receptor
JNK: c-Jun N-terminal kinase
PCOS: Polycystic ovary syndrome
FN: Fibronectin
CTGF: Connective tissue growth factor
