# Supplementary material for: Metformin protects against cyclophosphamide-induced ovarian fibrosis by MIF/CD74-mediated macrophage polarization
Source: J Transl Med. 2025 Nov 12;23:1273. doi: 10.1186/s12967-025-07294-5 (PMC12613643; doi:10.1186/s12967-025-07294-5)
Supplement: Supplementary file 2 — Supplementary Material 2 [file 12967_2025_7294_MOESM2_ESM.docx]

**Table S2** Antibodies used for immunostaining, immunofluorescence and western blot.

| **Antibodies** | **Identififier** | **Source** |
| --- | --- | --- |
| Rabbit anti-FSHR | 22665-1-AP | Proteintech |
| Mouse anti-AMH | PA5-35851 | Invitrogen |
| Rabbit anti-Fibronectin | ab2413 | Abcam |
| Rabbit anti-Collagen I | ab34710 | Abcam |
| Rabbit anti-CTGF | 10095 | Cell Signaling Technology |
| Rabbit anti-TGF-β1 | 3711 | Cell Signaling Technology |
| Rabbit anti-p38 MAPK | 9212 | Cell Signaling Technology |
| Rabbit anti-Phospho-p38 MAPK (Thr180/Tyr182) | 9211 | Cell Signaling Technology |
| Rabbit anti- Phospho-NF-κB p65(Ser536) | 3031 | Cell Signaling Technology |
| Mouse anti-NF-κB p65(Ser536) | sc-8008 | SANTA CRUZ |
| Rabbit anti-Phospho-IκB-α | 2859 | Cell Signaling Technology |
| Rabbit anti-IκB-α | 9242 | Cell Signaling Technology |
| Rabbit anti-MIF | 88186 | Cell Signaling Technology |
| Rabbit anti-CD74 | 77274 | Cell Signaling Technology |
| Rabbit anti-CD86 | ab239075 | abcam |
| Rabbit anti-CD206 | 18704-1-AP | Proteintech |
| Rabbit anti-F4/80 | 28463-1-AP | Proteintech |
| Rabbit anti-JNK1/2/3 | 44-682G | Invitrogen |
| Rabbit anti- Phospho-JNK1/2/3 | MA5-51555 | Invitrogen |
| Mouse anti-beta-Actin | T0022 | Affinity Biosciences |

**Table S3** Specific primers.

| **CD86** | Forward:5′- CCTTCCTGCTCTCTGCTAACTT -3′;  Reverse:5′- TAGGTTCTGGGTAACCGTGT -3′ |
| --- | --- |
| **CD206** | Forward:5′- GCCTCGTTGTTTTGCGTCTT -3′;  Reverse:5′- GAGAACAGCACCCGGAATGA -3′ |
| **CD74** | Forward:5′- ACCAAGTATGGCAACATGACAGA -3′;  Reverse:5′- AGTGGCGGGTACACCTTCAG -3′ |
| **MIF** | Forward:5′- TGCACAGCATCGGCAAGAT -3′;  Reverse:5′-AATAGTTGATGTAGACCCTGTCC -3′ |
| **COL1A1** | Forward:5′- CGTGTGCACCGCCAAAGAT -3′;  Reverse:5′- GTCTGGGCCAAACGTGTCTT -3′ |
| **TGF-β1** | Forward:5′- CAGAAATACAGCAACAATTCCTGG-3′;  Reverse:5′- TTGCAGTGTGTTATCCGTGCTGTC-3′ |
| **β-actin** | Forward:5′- GTTGGAGCAAACATCCCCCA -3′;  Reverse:5′- CTCAGACCTGGGCCATTCAG-3′ |
| **Ad-CD74** | Forward:5′- CGCAAATGGGCGGTAGGCGTG -3′;  Reverse:5′- GAAATTTGTGATGCTATTGC -3′ |
| **siCD74-1** | 5′-GCGACCTTATCTCCAACAA-3′ |
| **siCD74-2** | 5′-CCAAGCCTGTGAGCAAGAT-3′ |
| **siCD74-3** | 5′-GCCACCAAGTATGGCAACA-3′ |
