# Supplementary material for: Metformin protects against cyclophosphamide-induced ovarian fibrosis by MIF/CD74-mediated macrophage polarization
Source: J Transl Med. 2025 Nov 12;23:1273. doi: 10.1186/s12967-025-07294-5 (PMC12613643; doi:10.1186/s12967-025-07294-5)
Supplement: Supplementary file 4 — Supplementary Material 4 [file 12967_2025_7294_MOESM4_ESM.pdf]

Figure 1C

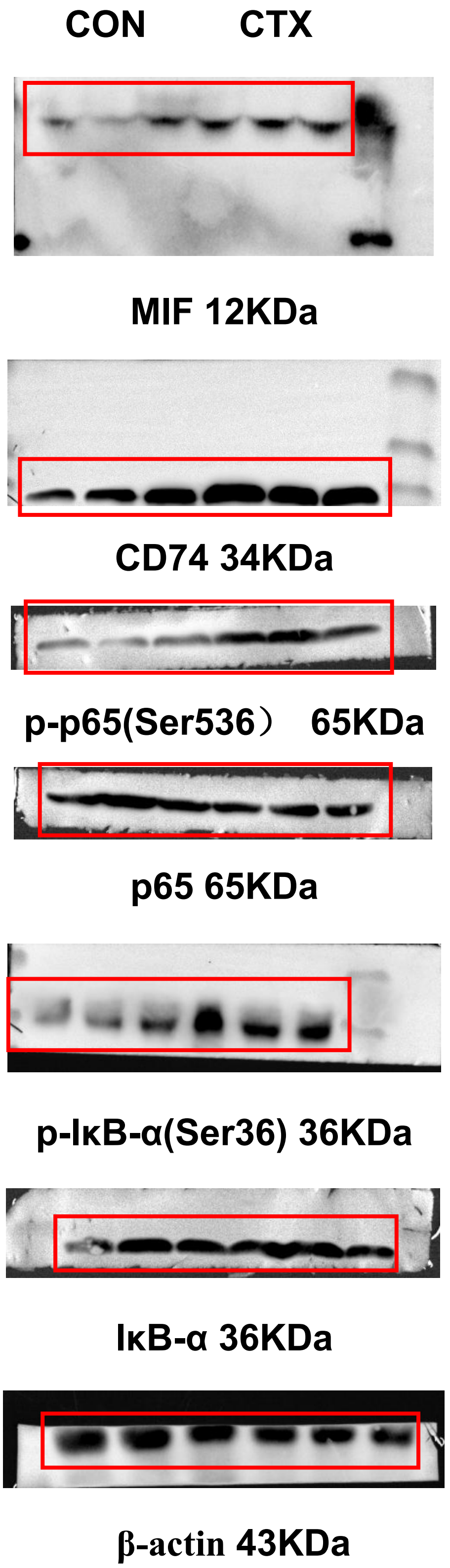
CON
CTX
MIF 12KDa
CD74 34KDa
p-p65(Ser536） 65KDa
p65 65KDa
p-IκB-α(Ser36) 36KDa
IκB-α 36KDa
β-actin 43KDa

Figure 1F

CON CTX

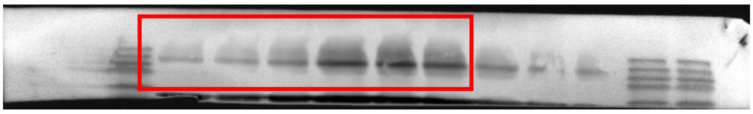

COL1A1 139KDa

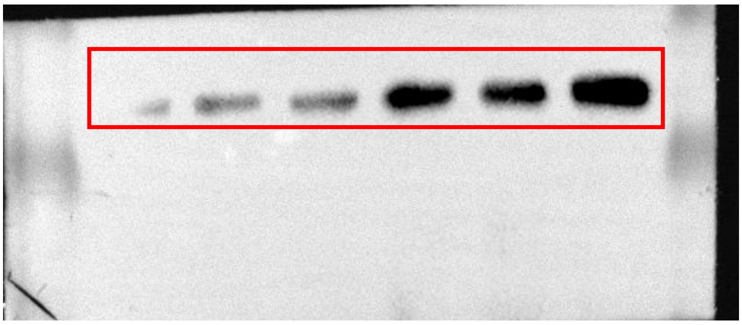

CTGF 38KDa

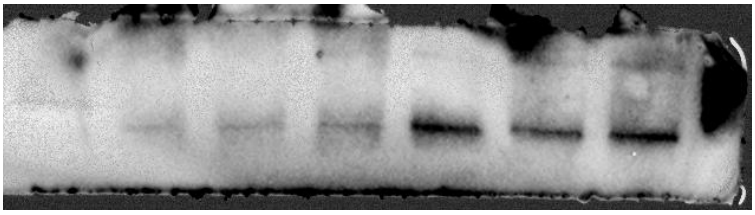

FN1 285KDa

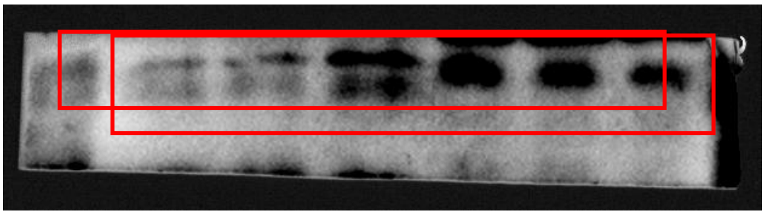

TGF-β1 33KDa

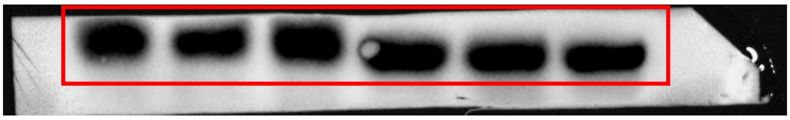

β-actin 43KDa

Figure 1H

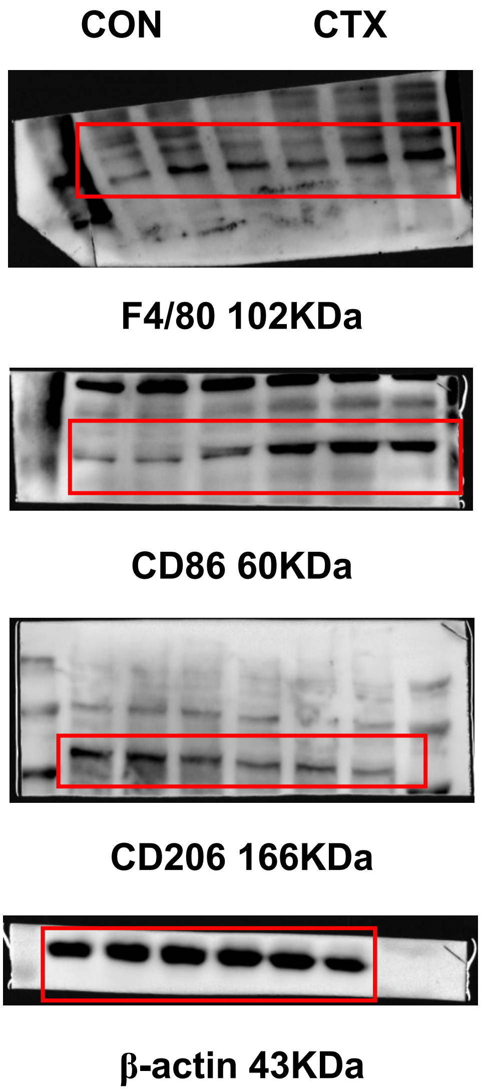

Figure 3C

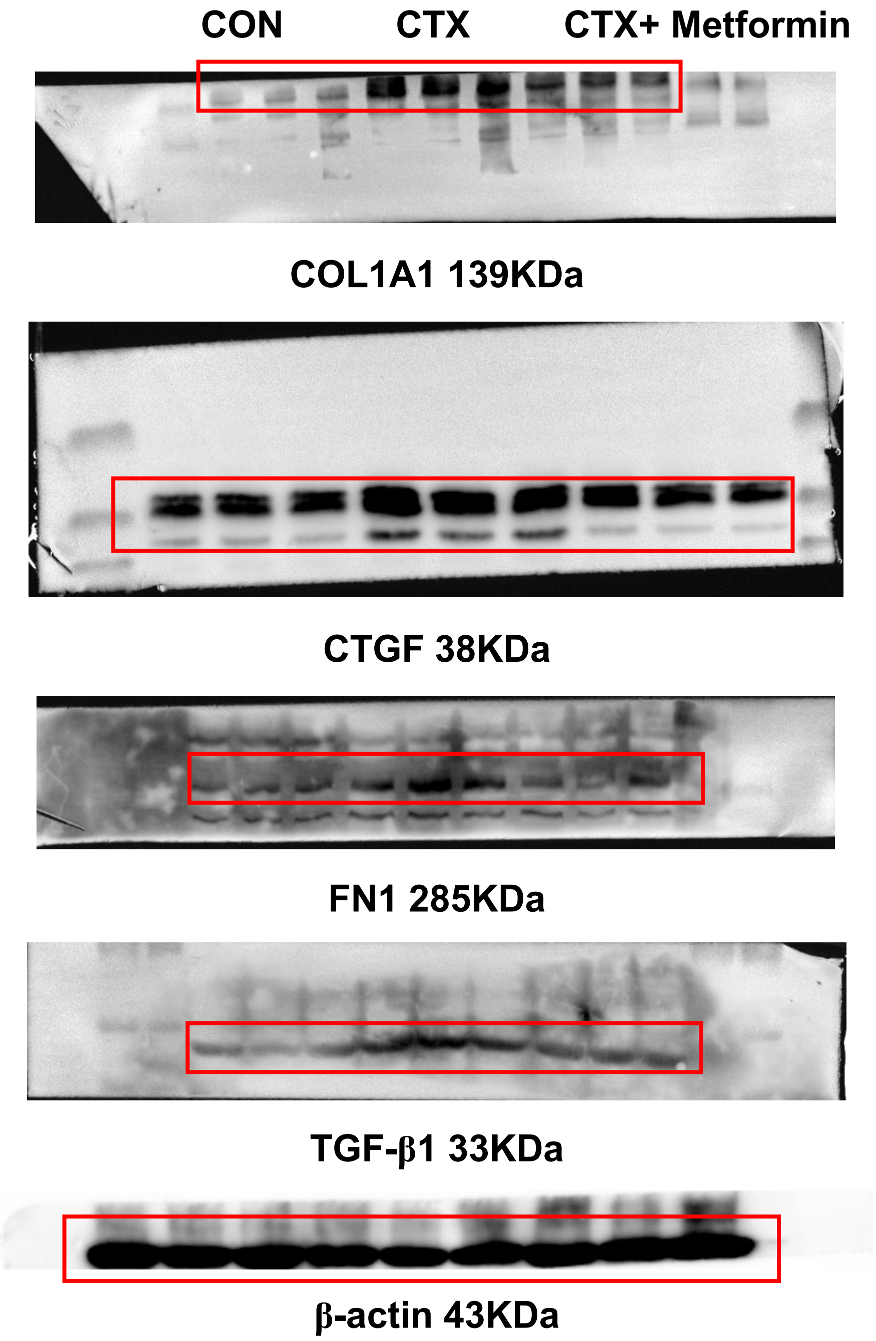
CON
CTX
CTX+ Metformin
COL1A1 139KDa
CTGF 38KDa
FN1 285KDa
TGF-β1 33KDa
β-actin 43KDa

Figure 3E

CON CTX CTX+ Metformin

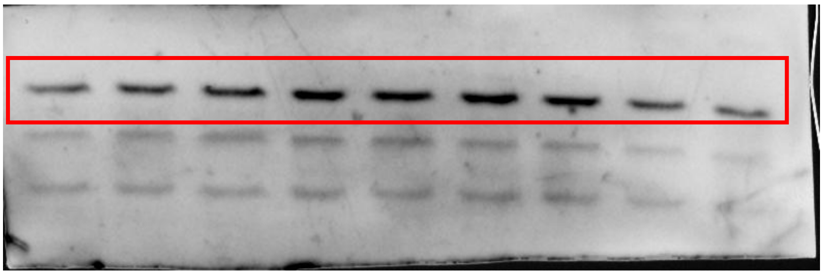

p-p38 43KDa

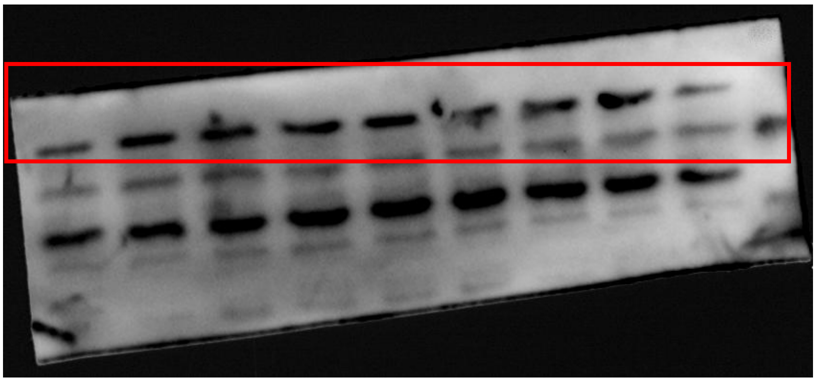

p38 43KDa

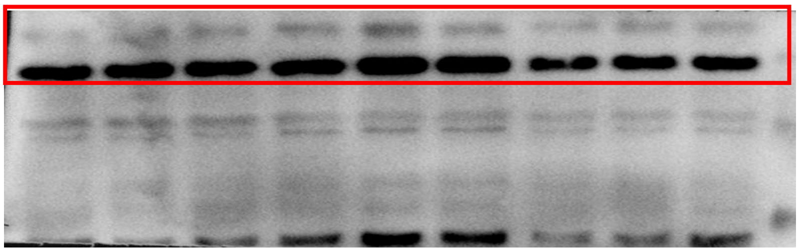

P-c-JNK 46/54KDa

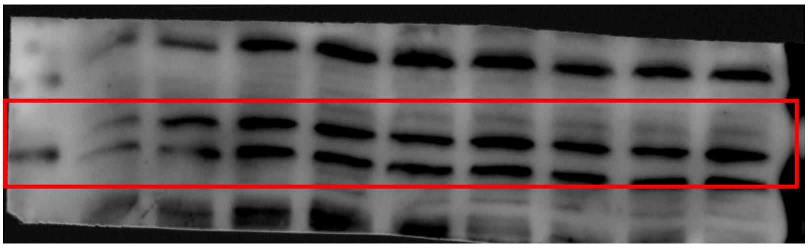

c-JNK 46/54KDa

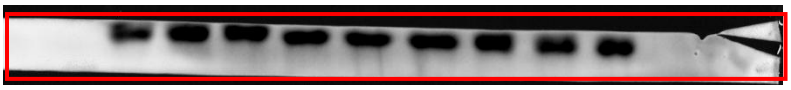

β-actin 43KDa

Figure 4G

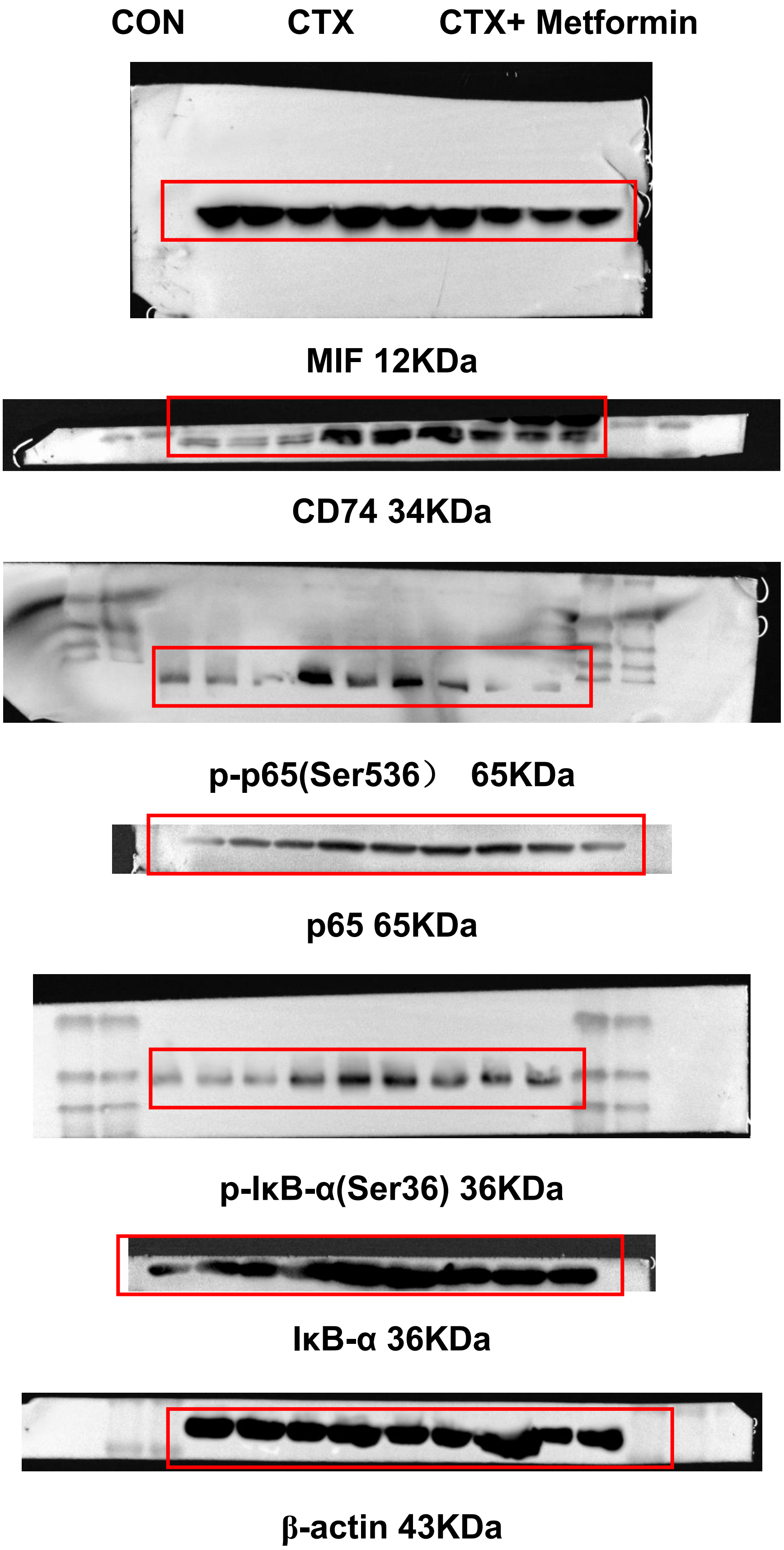
CON
CTX
CTX+ Metformin
MIF 12KDa
CD74 34KDa
p-p65(Ser536） 65KDa
p65 65KDa
p-IκB-α(Ser36) 36KDa
IκB-α 36KDa
β-actin 43KDa

Figure 5C

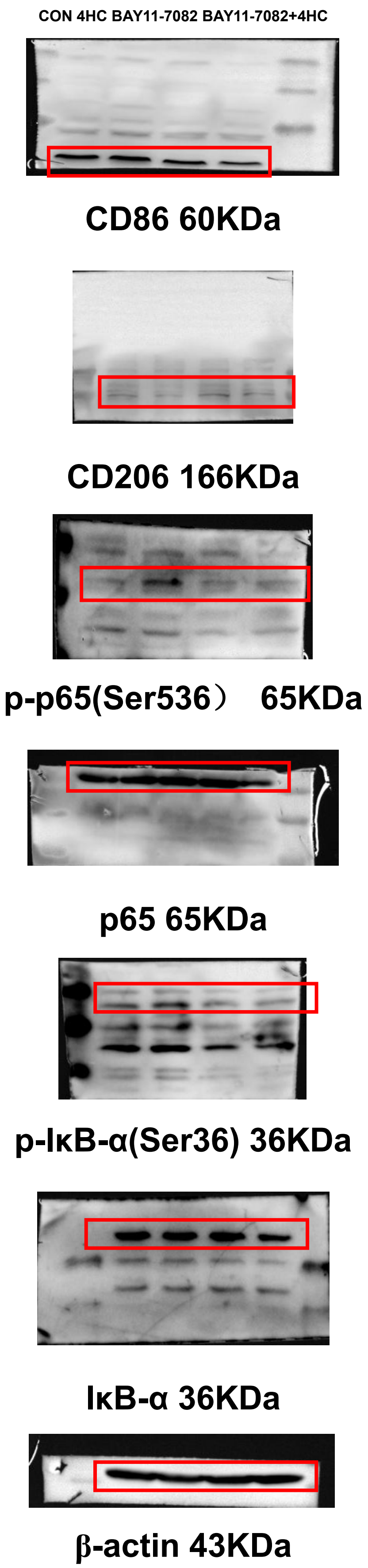
CON 4HC BAY11-7082 BAY11-7082+4HC
CD86 60KDa
CD206 166KDa
p-p65(Ser536） 65KDa
p65 65KDa
p-IκB-α(Ser36) 36KDa
IκB-α 36KDa
β-actin 43KDa

Figure 5J

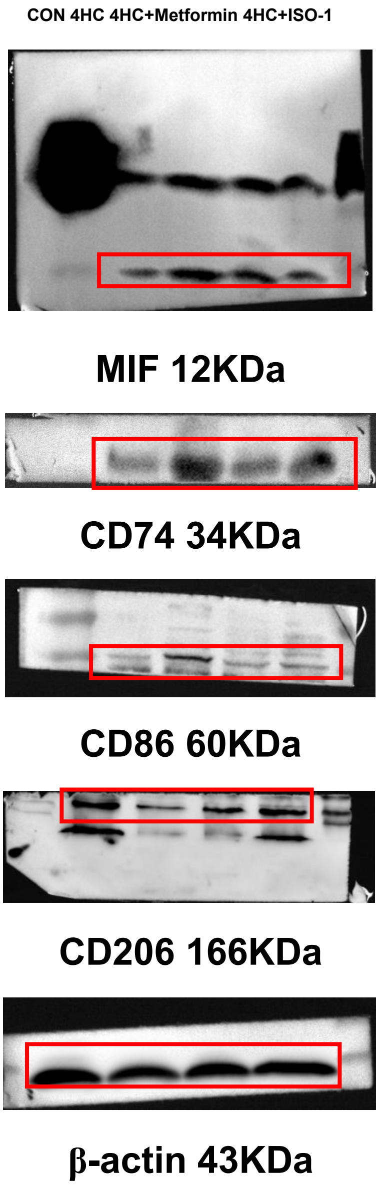
CON 4HC 4HC+Metformin 4HC+ISO-1
MIF 12KDa
CD74 34KDa
CD86 60KDa
CD206 166KDa
β-actin 43KDa

Figure 5J

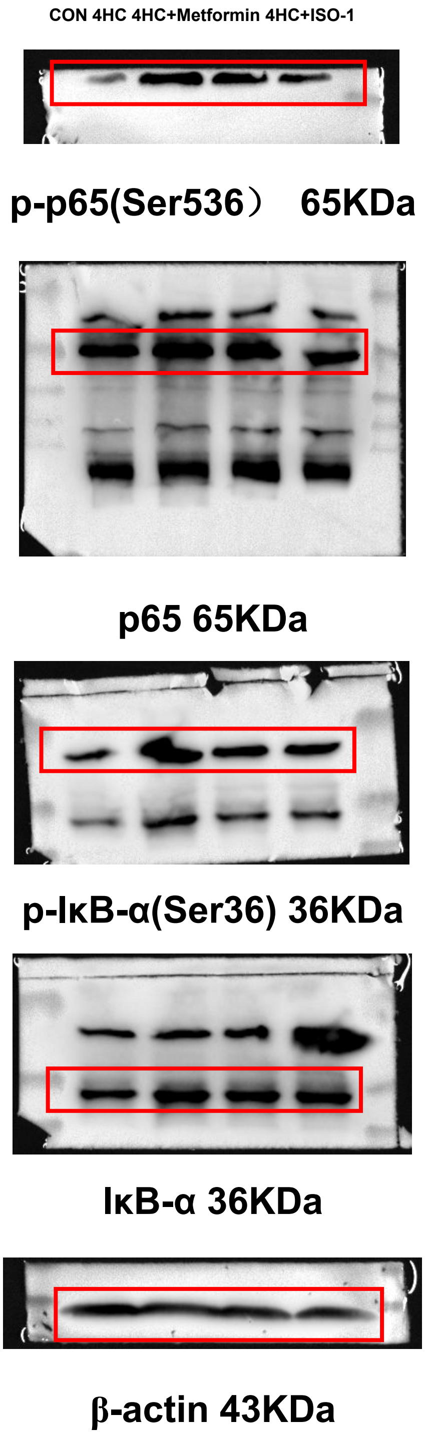
CON 4HC 4HC+Metformin 4HC+ISO-1
p-p65(Ser536） 65KDa
p65 65KDa
p-IκB-α(Ser36) 36KDa
IκB-α 36KDa
β-actin 43KDa

Figure 6E

KGN KGN+HSF

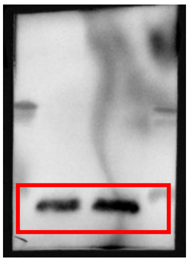

PCNA 36KDa

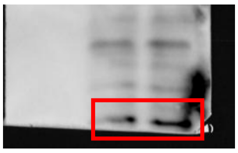

AMH 60KDa

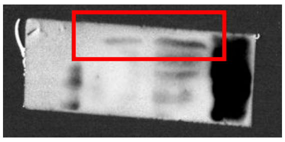

FSHR 78KDa

Figure 6J

HSF HSF+THP1 HSF+THP1+ISO-1

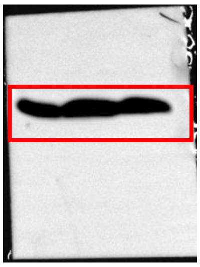

MIF 12KDa

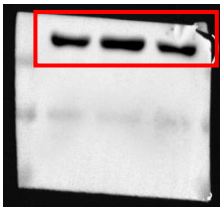

CD74 34KDa

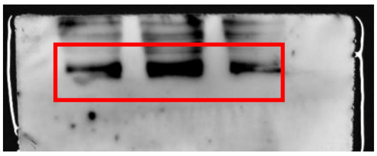

COL1A1 139KDa

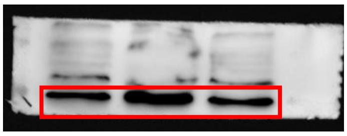

CTGF 38KDa

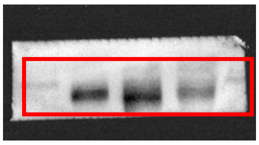

FN1 285KDa

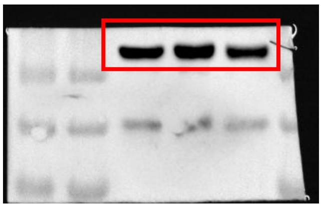

TGF-β 1 33KDa

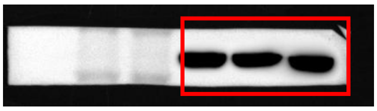

β -actin 43KDa

Figure 6K

CON THP1 SB203580 THP1+SB203580

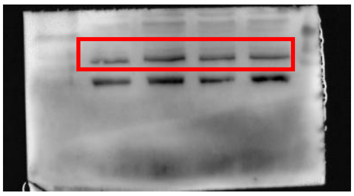

COL1A1 139KDa

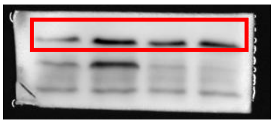

CTGF 38KDa

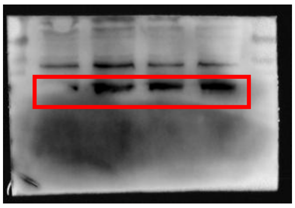

FN1 285KDa

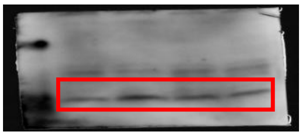

TGF-β1 33KDa

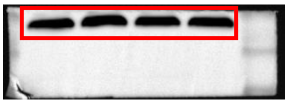

β-actin 43KDa

Figure 6K

CON THP1 SB203580 THP1+SB203580

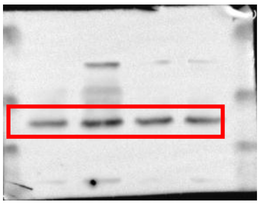

p-p38 43KDa

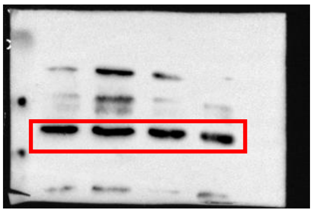

p38 43KDa

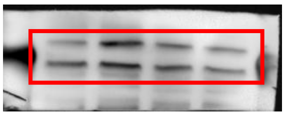

P-c-JNK 46/54KDa

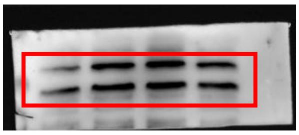

c-JNK 46/54KDa

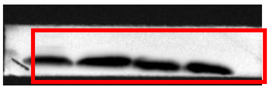

β-actin 43KDa

Figure 7B

Control si-CD74 si-NC

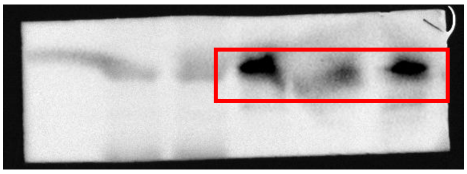

CD74 34KDa

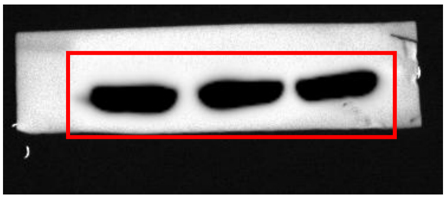

β-actin 43KDa

Figure 7E

Control si-CD74 si-NC

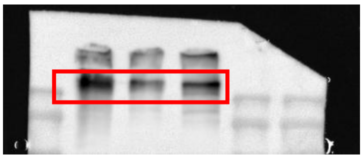

COL1A1 139KDa

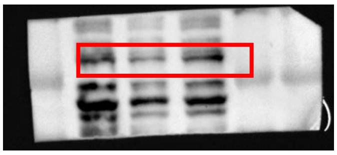

CTGF 38KDa

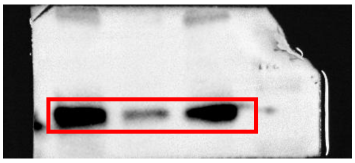

FN1 285KDa

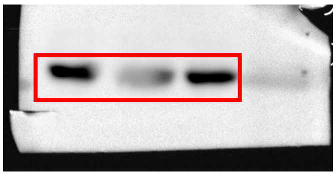

TGF-β 1 33KDa

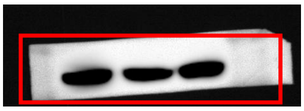

β-actin 43KDa

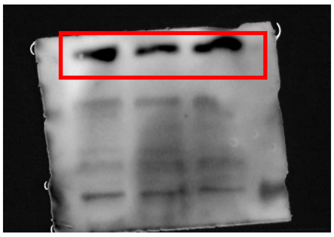

p-p38 43KDa

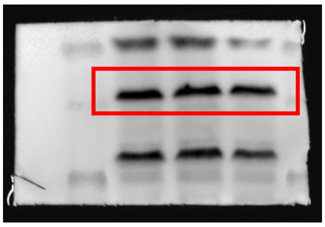

p38 43KDa

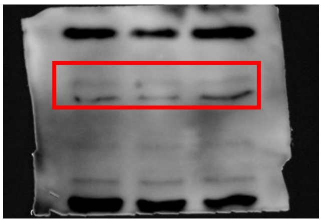

P-c-JNK 46/54KDa

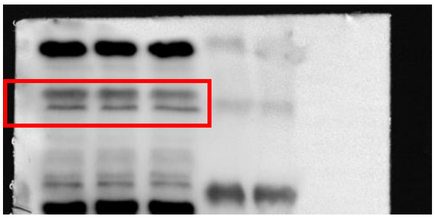

c-JNK 46/54KDa

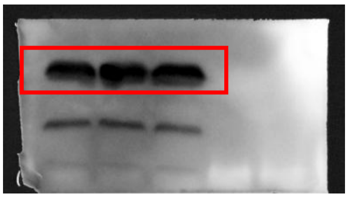

β-actin 43KDa

Figure 7H

Control Ad-CD74 Ad-NC

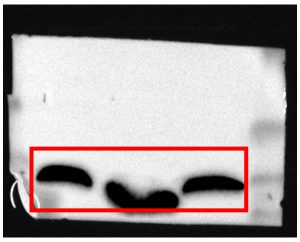

CD74 34KDa

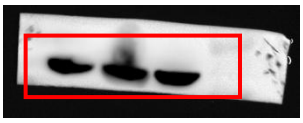

β-actin 43KDa

Figure 7K

Control Ad-CD74 Ad-NC

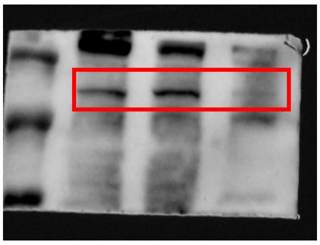

COL1A1 139KDa

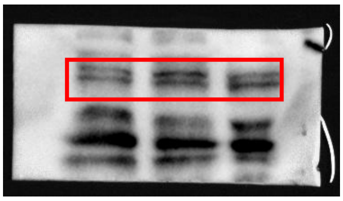

CTGF 38KDa

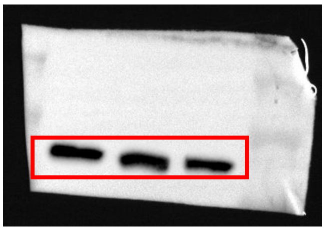

FN1 285KDa

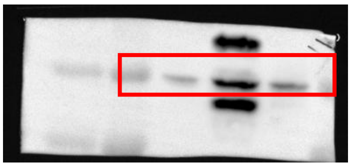

TGF-β 1 33KDa

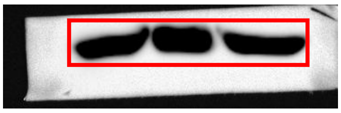

β-actin 43KDa

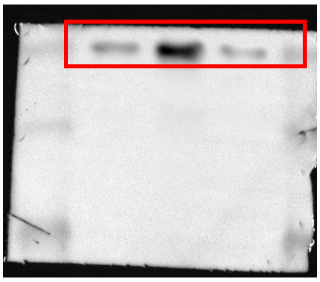

p-p38 43KDa

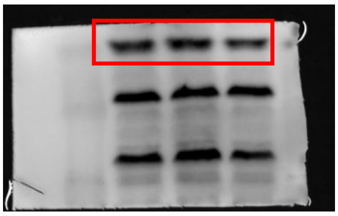

p38 43KDa

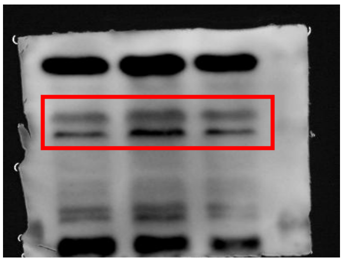

P-c-JNK 46/54KDa

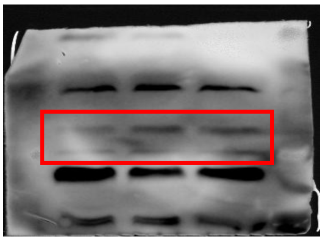

c-JNK 46/54KDa

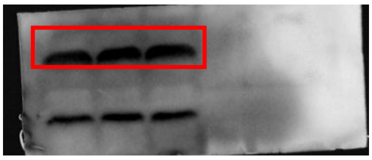

β-actin 43KDa
