# Supplementary material for: Metformin protects against cyclophosphamide-induced ovarian fibrosis by MIF/CD74-mediated macrophage polarization
Source: J Transl Med. 2025 Nov 12;23:1273. doi: 10.1186/s12967-025-07294-5 (PMC12613643; doi:10.1186/s12967-025-07294-5)
Supplement: Supplementary file 5 — Supplementary Material 5 [file 12967_2025_7294_MOESM5_ESM.pdf]

A

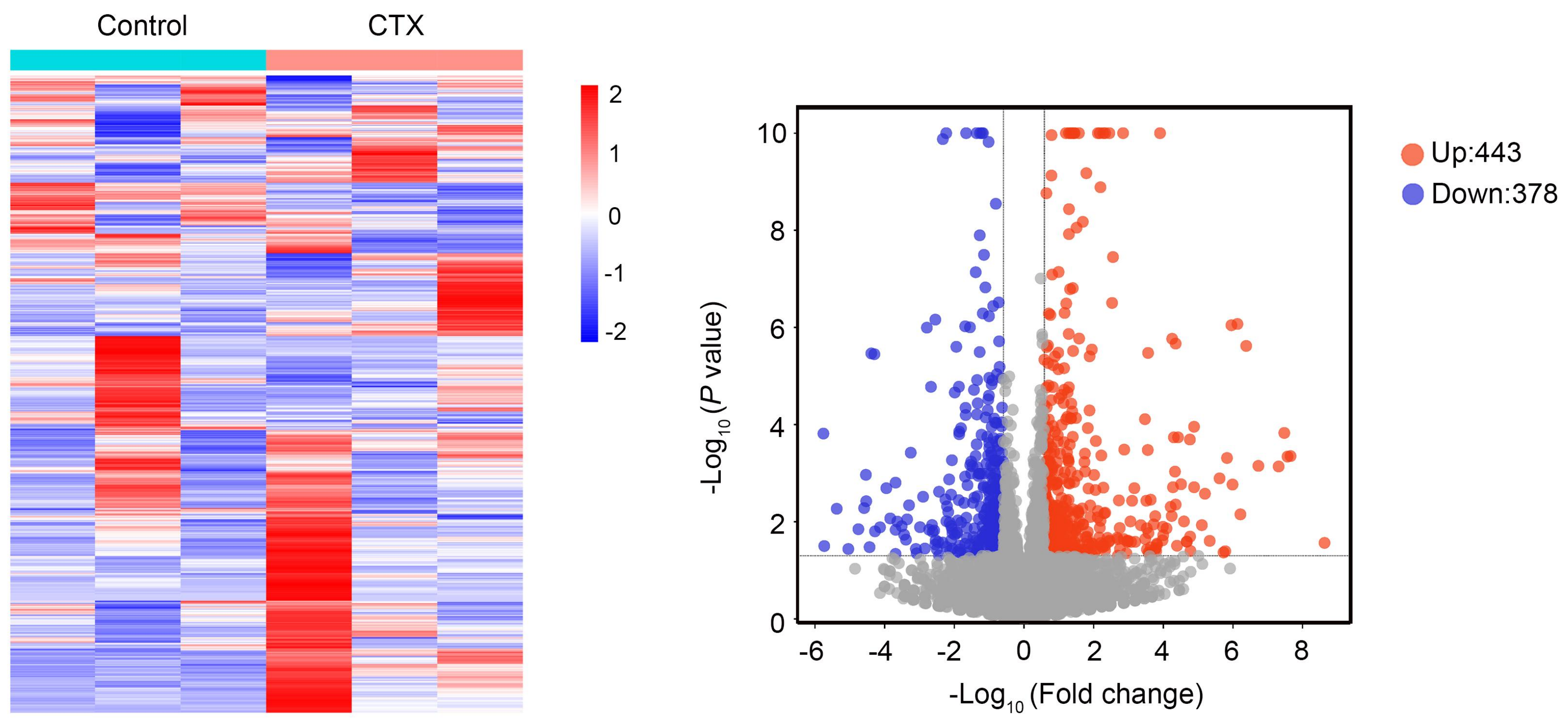

Supplemental Figure S1. DEG enrichment analysis of the ovaries of mice treated with control and CTX-treated mice (n=3 mice per group). Heatmaps showing the DEGs. DEGs, differentially expressed genes.

A

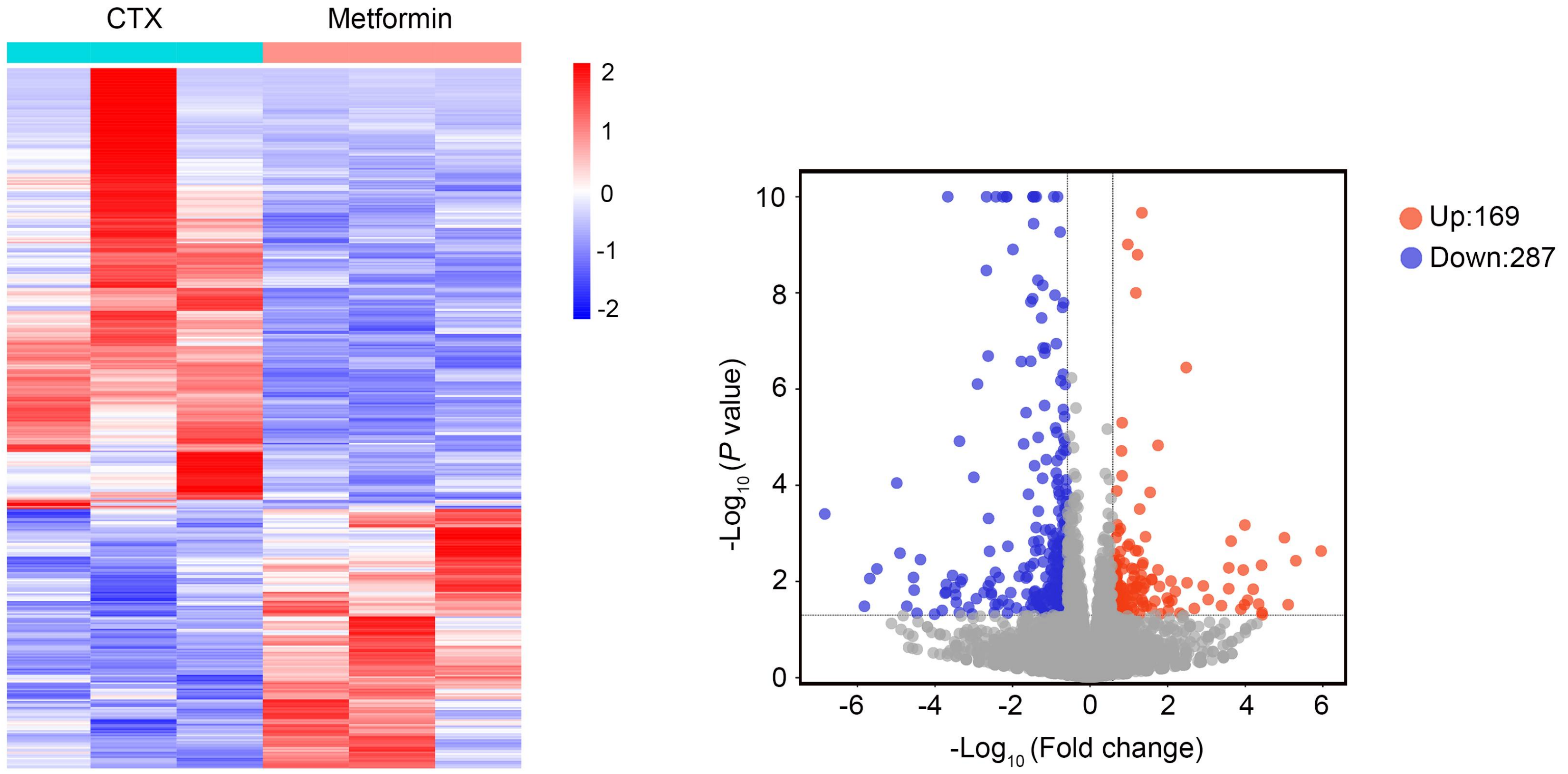

Supplemental Figure S2. DEG enrichment analysis of the ovaries of mice treated with CTX alone or CTX+ metformin mice (n=3 mice per group). Heatmaps showing the DEGs. DEGs, differentially expressed genes.

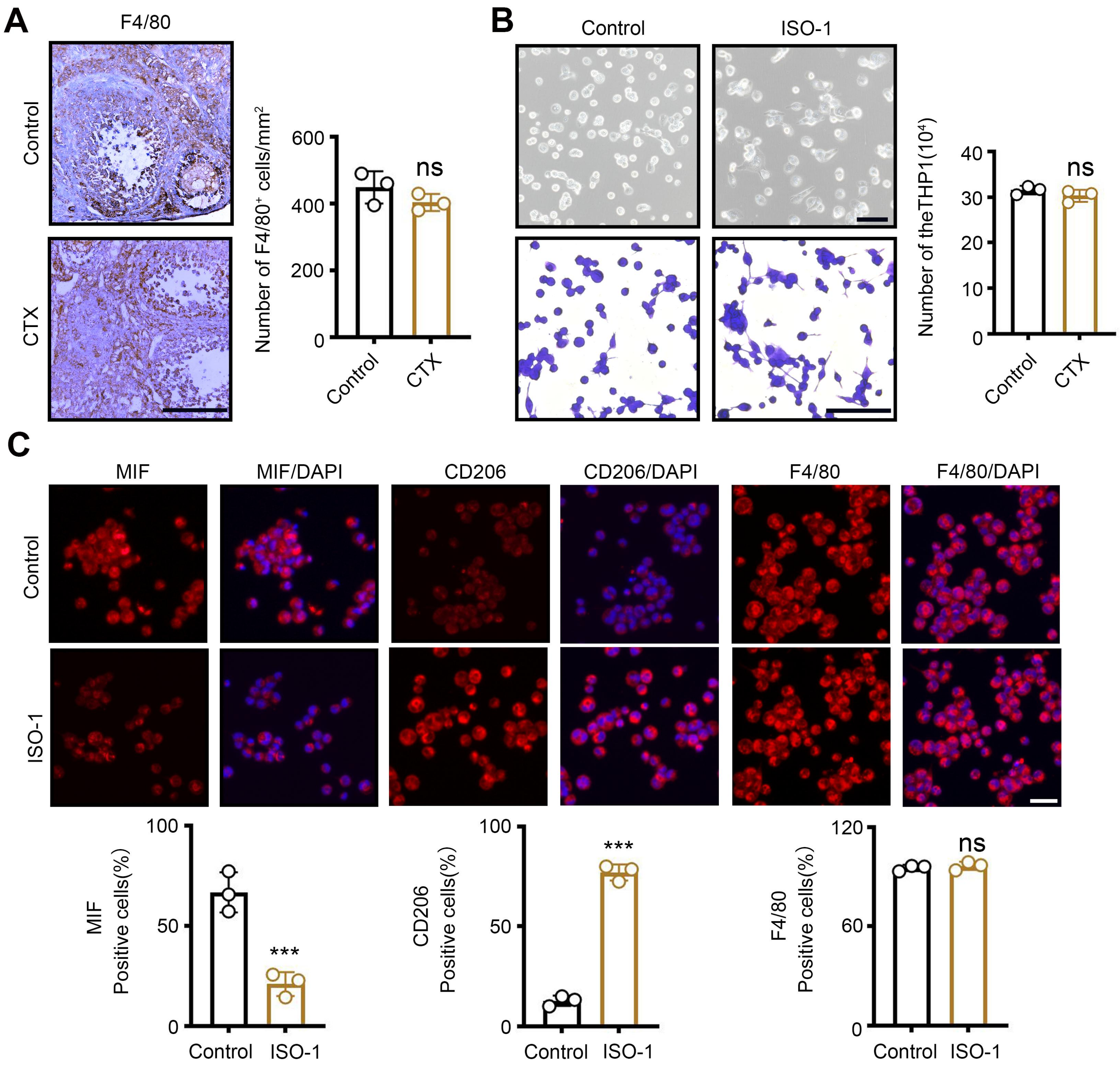

Supplemental Figure S3. The effect of MIF on macrophage recruitment and polarization. (A) Immunostaining for F4/80 expression in ovaries of CTX-treated and control ovaries. (B) Morphological changes in THP-1 Cells treated with the ISO-1(20μM) for 24 h. (B) Count of macrophage number. (C) Immunofluorescence staining for MIF, CD206 and F4/80 expression. Scar bar=100 μm. Scale bars: 100 μm. P>0.05 and ***P<0.001 for the indicated comparisons

A

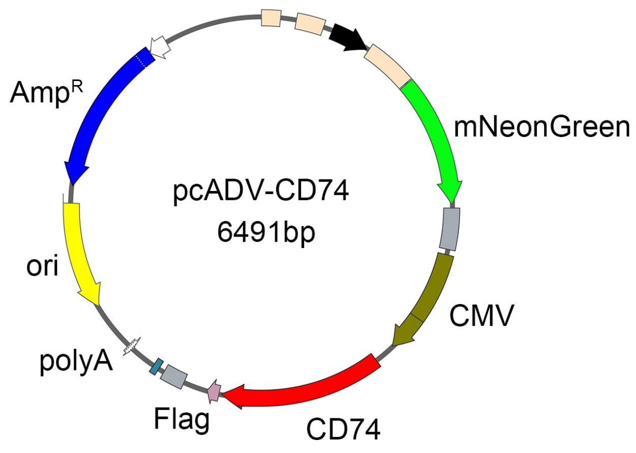

Supplemental Figure S4. Viral expression vector backbone for overexpressing CD74.
